# Photophysical and Bactericidal Properties of Pyridinium and Imidazolium Porphyrins for Photodynamic Antimicrobial Chemotherapy

**DOI:** 10.3390/molecules26041122

**Published:** 2021-02-20

**Authors:** Florent Le Guern, Tan-Sothéa Ouk, Issabayev Yerzhan, Yesmurzayeva Nurlykyz, Philippe Arnoux, Céline Frochot, Stéphanie Leroy-Lhez, Vincent Sol

**Affiliations:** 1 Institut Lavoisier de Versailles, Université Paris-Saclay, UVSQ, CNRS, 78035 Versailles, France; florent.le-guern@uvsq.fr; 2 Laboratoire PEIRENE, Université de Limoges, EA 7500, 123 Avenue Albert Thomas, 87060 Limoges CEDEX, France; tan-sothea.ouk@unilim.fr (T.-S.O.); stephanie.lhez@unilim.fr (S.L.-L.); 3 Laboratoire Réactions et Génie des Procédés (LRGP), Université de Lorraine, UMR 7274 CNRS, ENSIC, 1 rue Grandville, 54000 Nancy, France; issabaev@gmail.com (I.Y.); yesmurzayeva.nurly@gmail.com (Y.N.); philippe.arnoux@univ-lorraine.fr (P.A.); celine.frochot@univ-lorraine.fr (C.F.)

**Keywords:** photodynamic, therapy, antimicrobial, cationic, porphyrin, properties, photophysical

## Abstract

Despite advances achieved over the last decade, infections caused by multi-drug-resistant bacterial strains are increasingly becoming important societal issues that need to be addressed. New approaches have already been developed in order to overcome this problem. Photodynamic antimicrobial chemotherapy (PACT) could provide an alternative to fight infectious bacteria. Many studies have highlighted the value of cationic photosensitizers in order to improve this approach. This study reports the synthesis and the characterization of cationic porphyrins derived from methylimidazolium and phenylimidazolium porphyrins, along with a comparison of their photophysical properties with the well-known *N*-methylpyridyl (pyridinium) porphyrin family. PACT tests conducted with the tetracationic porphyrins of these three families showed that these new photosensitizers may offer a good alternative to the classical pyridinium porphyrins, especially against *S.aureus* and *E.coli*. In addition, they pave the way to new cationic photosensitizers by the means of derivatization through amide bond formation.

## 1. Introduction

Since the introduction of penicillin in the 1940s, the era of antibiotics has been considered as miraculous, given that these treatments had significantly decreased the number of deaths resulting from bacterial infections [1]. Despite the well-known rise in antibiotic-resistant bacteria, these drugs have been wrongly used all around the world, due in particular to faulty communications and common beliefs [2]. In addition to antibioresistance, leading to the emergence of superbugs or multidrug-resistant (MDR) strains, the pace of new antibiotic discoveries has gradually slowed down in the recent years [3,4]. The issues due to microbial resistance are currently recognized worldwide by health organizations [5]. Consequently, alternative approaches have been investigated to reduce the impact of microbial resistance.

Around the world, laboratories have focused their attention on a recent and promising alternative to antibiotics, namely photodynamic antimicrobial chemotherapy (PACT). This technique relies on the light activation of photosensitizers (PS) in the presence of molecular oxygen (^3^O_2_). Under appropriate light irradiation, PS activation leads to the production of singlet oxygen (^1^O_2_) by energy transfer, or oxygen radicals (such as superoxide anion O_2_^•−^, hydroxyl radical HO^•^, hydroperoxyl radical HOO^•^) by electron transfer. These reactive oxygen species (ROS) are able to rapidly react with a large panel of molecules (proteins, lipids, nucleic acids, metabolites) and cause cellular damages, which ultimately lead to cell death. The number and diversity of the cellular targets greatly limit the rise in bacterial strains that resist this treatment [6,7]. Owing to their very short life span, ROS do not end up in the waste system unlike common sanitizers, and are thus environmentally friendly [6,8]. Although PACT exhibits many advantages for bacterial photoinactivation, its lack of specificity is a major drawback, as it may cause damages to the host tissues as well [9,10,11]. PACT was first tried out against methicillin-resistant *S. aureus* (MRSA), with promising results [12]. Unfortunately, Gram-negative bacteria have shown a poor sensitivity to the PS initially studied, which has been attributed the protection provided by their outer membrane [13,14]. Nevertheless, it has been shown that cationic PS exhibited a good affinity for bacteria by interacting through electrostatic interactions, with the highly negatively charged cell wall components such as lipoteichoic acid (LTA) and lipopolysaccharide (LPS) in Gram-positive and Gram-negative bacteria, respectively [15,16,17]. Accordingly, cationic porphyrins displayed photoinduced activity against a broad spectrum of bacteria [17,18,19,20], and this trend was also confirmed with several families of cationic PSs [21,22,23].

As members of the tetrapyrrolic PS, these compounds can be classified according to the functions substituted at the *meso* positions: A_4_ porphyrins are related to symmetric and uniform compounds, A_3_B to asymmetric compounds with one *meso* position substituted by a different function, and A_2_B_2_ to porphyrins equally substituted by two different functions (*cis* or *trans* isomers) [24]. Unfortunately, a lack of data comparing the photophysical and photobactericidal properties of cationic porphyrins may be problematic at the beginning of a study. Except for aryl porphyrins carrying ammonium derivatives [25,26,27], these cationic PS are mostly obtained by using *N*-methylated pyridyl (also called pyridinium) groups [16,17,18,28,29]. Pyridinium porphyrins, as well as aryl porphyrin-carrying ammonium derivatives, are well-known in PACT as these PS have shown very good activities against several strains. As their efficacy is partially due to their quaternary ammonium functions [17], porphyrins carrying imidazole groups (or *N*-methyl derivatives) should also show interesting properties. Moreover, even if imidazole (metallo)porphyrins have been studied for their conductivity properties [30,31,32] or their antioxidant potencies [33], their photocytotoxicity has rarely been spotlighted [34,35], especially in PACT as A_4_ or A_3_B structures [36,37,38]. In the same way, phenylimidazole porphyrins have also been presented in few reports, but never as A_4_ porphyrins [39]. We report herein the synthesis, and photochemical and photophysical characterization of six different families of pyridinium and imidazolium porphyrins along with a comparison of their efficiency against Gram-positive and Gram- with a negative bacterial strains special underscoring on A3B porphyrins. In connection with our research studies in PACT [40,41,42,43,44], we have devised synthetic routes to obtain porphyrin derivatives, which have been designed to selectively target bacteria [45,46]. Through these studies, it occurred to us that amine or carboxylic acid functions are very important to build amide bonds between PS and the targeting moieties (such as peptides). Thus, these functions have been distributed among the families of porphyrins synthetized in this study (Table 1).

## 2. Results and Discussion

### 2.1. Synthesis and Characterization

**Figure 1:**
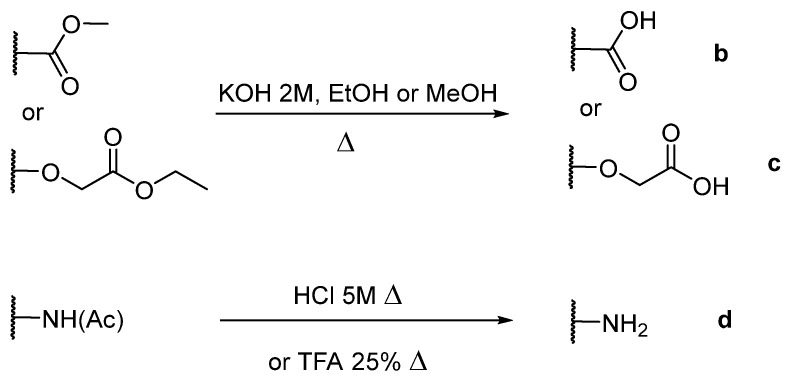
Deprotection of carboxylic acid and amine functions.

The porphyrins described in this study were synthesized using the Little [58] or Adler–Longo [59] protocols. These porphyrins belong to six families named after their *meso*-substituted groups: **1**: Pyridyl, **2**: *N*-methylpyridyl (or pyridinium), **3**: Phenylimidazole, **4**: Phenylimidazolium, **5**: Methylimidazole, and **6**: Methylimidazolium. In these groups, A_4_ porphyrins were first obtained (**1a** to **6a**). Then, in each main family, A_3_B porphyrins were synthesized to observe the consequence of different functions on their photophysical properties. Generally, these functions are introduced into the porphyrin structure by mixing a functionalized benzaldehyde with other aldehydes during the synthesis, following the Little protocol. In order to offer a global and efficient procedure for all porphyrins, only protected functionalized aldehydes have been used in these syntheses. These protections ease the purification steps, which are known to present difficulties. Indeed, the purification of porphyrins carrying a free carboxylic acid function mainly depends on the brand of the silica gel. Different functionalized benzaldehydes have been chosen to provide reactive groups after deprotection: Methyl 4-formylbenzoate for a carboxylic acid function (**b,** electron-withdrawing group (EWG)), ethyl 4-formylphenoxyacetate for a carboxylic acid function on a hydroxyl-based porphyrin (**c,** EDG), and 4-acetamidobenzaldehyde for a primary amine function (**d,** electron-donor group (EDG)) (Figure 1).

4-acetamidobenzaldehyde was chosen to obtain porphyrins carrying an amine function but, according to other studies, these compounds can also be obtained by using 4-nitrobenzaldehyde [60,61]. *N*-Methylated A_3_B porphyrins carrying one carboxylic acid function were directly prepared from the respective A_3_B porphyrins (**2b** from **1b**, for example), as the carboxylic group only reacts with iodomethane under its carboxylate form. To obtain the *N*-methylated A_3_B porphyrin **2d** carrying one primary amine, *N*-methylation had to been done before deprotection to avoid any methylation of the primary amine. Owing to the water-solubility of this porphyrin and the impossibility of washing out excess salts, HCl was replaced by TFA for the deprotection step as TFA can be totally removed by successive evaporation and freeze-drying steps. The different deprotection steps have been performed with satisfying yields. Finally, A_2_B_2_ porphyrins were also prepared to support prospective works using two coupling agents (**5e** and **6e**). Unfortunately, the separation of *trans* and *cis* isomers could not be achieved using common purification procedures. Therefore, all data concerning **5e** and **6e** are obtained by using an isomer mixture.

The porphyrin syntheses did present some yield fluctuations, depending on both the family and the functionalized aldehyde (Table 2). The synthesis of pyridyl porphyrins **1** is well-known from the literature, whereas phenylimidazole **3** and methylimidazole porphyrins **5** are less common in PACT. Unfortunately, the final yield of **5a** synthesis is lower than that reported in the literature (generally about 10–12%) because of an interaction of the compound with silica gel used for chromatography. Thus, alumina is usually advised for such compounds [34]. **3a** was the easiest porphyrin to purify because of the particularly high final yield. Whereas the formation of A_3_B porphyrins from **1** and **5** leads to similar yields, asymmetric porphyrins from **3** have been obtained with very good yields, which are three times higher than the average. Carboxylic porphyrins (**b** and **c**) have been recovered with similar results, but the use of 4-acetamidobenzaldehyde has induced a significant drop in yield. Furthermore, methyl 4-formylbenzoate seemed to be very reactive as the presence of all possible porphyrins was found in the crudes (B_4_, AB_3_, A_2_B_2_) despite the advised reactant proportions for A_3_B synthesis (3 eq of A, 1 eq for B).

The synthesized porphyrins exhibited varied solubility properties. Generally, phenylimidazole porphyrins **3** were less soluble than all other porphyrins, even if a high purity of porphyrin usually involves some solubility issues that can be resolved by adding minute amounts of methanol into usual apolar solvents. *N*-Methyl porphyrins are supposed to be water-soluble but a primary dissolution into a DMSO solution is strongly advised to avoid any incomplete dissolution. Due to the solubility issues, the analysis of porphyrins may appear difficult in classical solvent. To obtain NMR analysis of **1**, **3**, and **5**, a mixture with CDCl_3_ and CD_3_OD has been used that may induce some peak shifting and multiplication depending on the device used. Fortunately, all porphyrins could be identified by MS (ESI +) and ^1^H NMR (Supplementary Materials Figures S1 to S18). Due to the asymmetric methylimidazole group in **5**, different stereoisomers were actually obtained. Thus, NMR spectra of porphyrins **5** have shown some peak multiplication, especially for the signals attributed to *N*-methyl protons. The total *N*-methylation of the imidazole group, in porphyrin family **6**, led to symmetric signals that could indicate an even distribution of the positive charge within the imidazole ring.

**Figure 2:**
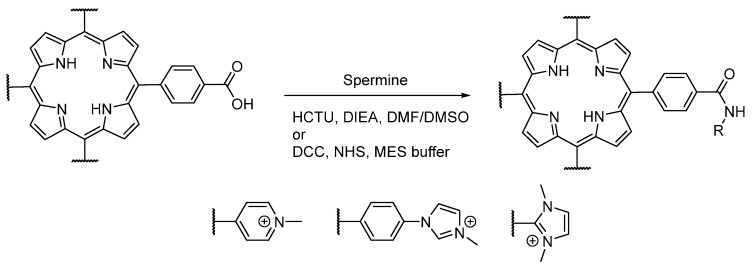
Investigation of carboxylic acid reactivities of cationic porphyrins using coupling agents.

The carboxylic acid reactivity of the different water-soluble porphyrins was also investigated (Figure 2). *N*-Methylation of **1b** (leading to **2b**) induced a drop in the reactivity of the carboxylic function. Indeed, **2b** has shown a poor reactivity in different conditions (buffers or DMSO), in spite of the use of different catalysts (carbodiimide or uronium salts). Thus, in order to obtain a conjugate based on **2a**, the coupling should be done before the *N*-methylation step, which implies that the new moiety should not react with iodomethane afterward. Otherwise, the carboxylic acid of **1b** should be activated by coupling an *N*-hydroxysuccinimide group before *N*-methylation [49]. On the other hand, other porphyrins (**4b**, **4c**, **6b**, **6e**) were able to react with these catalysts, leading then to the formation of an amide function in the presence of a primary amine.

### 2.2. Photophysical Properties

**Figure 3:**
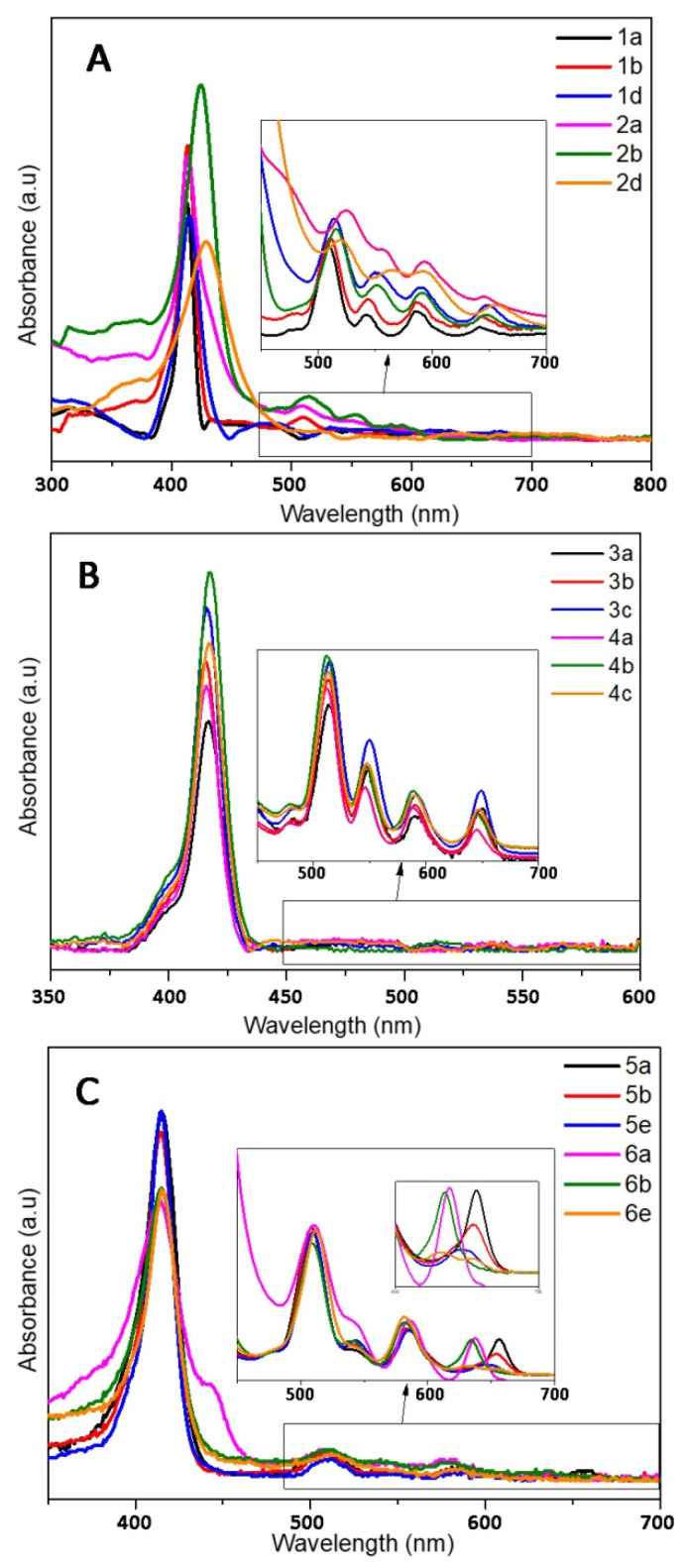
Absorption spectra of the different porphyrins in ethanol: (**A**) **1** and **2**, (**B**) **3** and **4**, (**C**) **5** and **6**.

**Figure 4:**
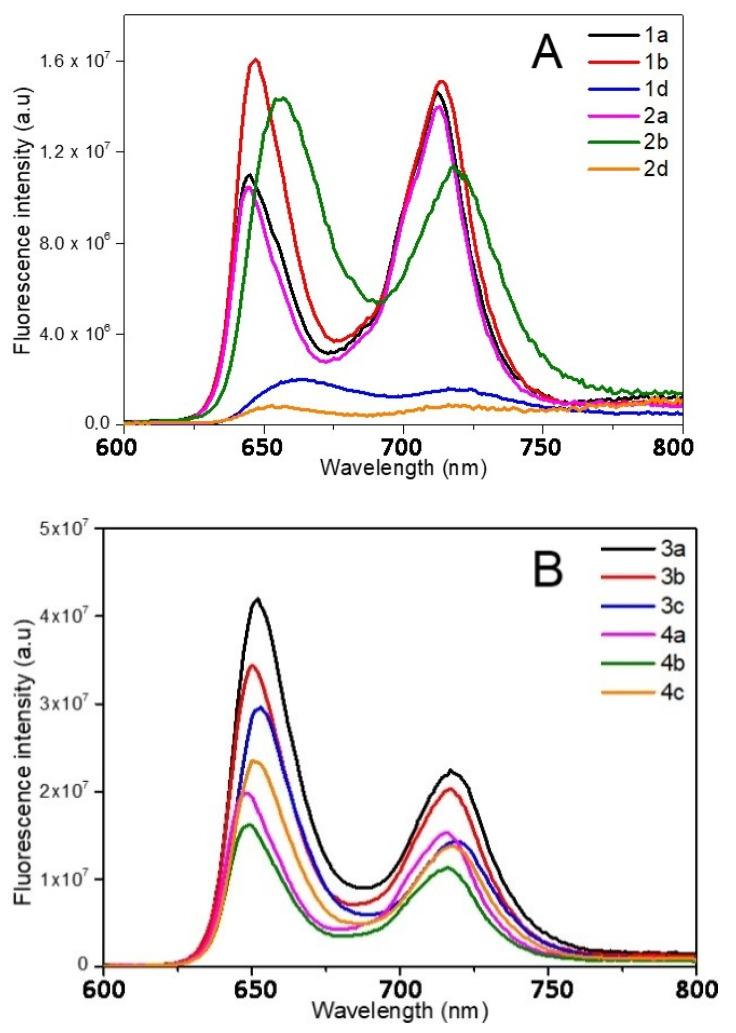

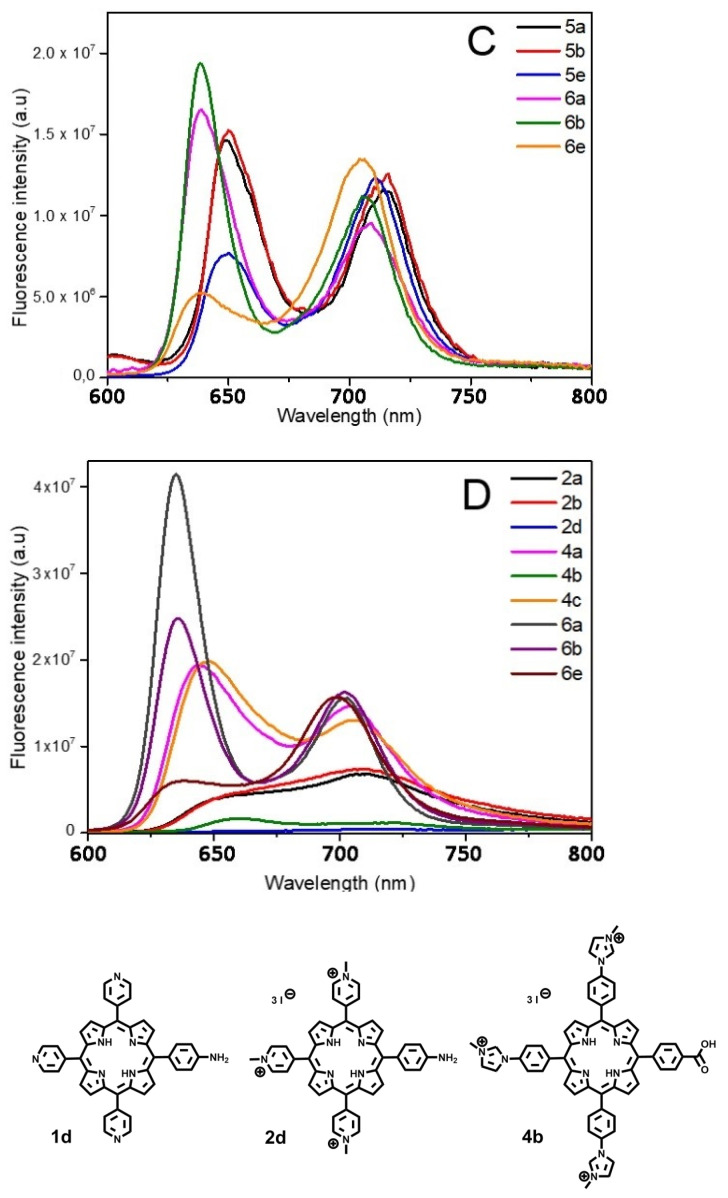
Fluorescence spectra of the different porphyrins in ethanol: (**A**) **1** and **2**, (**B**) **3** and **4**, (**C**) **5** and **6**. In deuterium oxide: (**D**) **2**, **4**, and **6** (λ_exc_ = 414 nm). Structures of quenched porphyrins are also represented (only in D_2_O for **4b**).

The photophysical properties of the different porphyrins have been investigated in ethanol (Figure 3 and Figure 4 and Table 3 and Table 4). In order to correlate the following biological trials, the photophysical properties of cationic porphyrins had also been investigated through further studies in aqueous solution (D_2_O).

Despite a few discrepancies due to the poor solubility of pyridinium porphyrins in ethanol, absorption spectra of **1** and **2** are fairly similar (Figure 3). Indeed, the main distinctions are due to aggregation phenomena that can be observed through the broadening of the Soret band [62]. On the opposite, florescence emission is greatly influenced by the kind of substituent (Figure 4A). Surprisingly, fluorescence spectra of **1a** and **2a** are similar in ethanol, which indicate that the different substituents (pyridyl or *N*-methylpyridinium) have no significant influence on the excited state properties of these compounds in this solvent. Lifetimes are also consistent with the usual data of porphyrins (*ca*. 10 ns). However, the Q (0,0) band of porphyrins **1a** and **2a** is less intense than the Q (0,1) band (ratio Q(0,0)/Q(0,1) < 1), which differs from the usual fluorescence emission spectra of porphyrins (Table 4). According to Vergeldt and co-workers [63], this discrepancy is due to the electron-accepting properties of pyridyl groups that have a direct influence on the Q (0,0) bands. Thus, the presence of a carboxylic acid group, instead of a pyridyl or pyridinium group in **1b** and **2b,** respectively, leads to a ratio Q (0,0)/Q (0,1) > 1. Moreover, a red-shift in the fluorescence emission of **2b** is observed, which is coherent with its absorption spectrum. In D_2_O, in addition to a lower fluorescence quantum yield, the ratio Q (0,0) / Q (0,1) dropped below 1 for **2b**, and the emission spectrum of this compound then became very similar to the well-known structureless broad band found in the spectrum of **2a** in D_2_O [63]. As the fluorescence emission properties of **2a** and **2b** are similar in D_2_O, it is likely that, in this solvent, the carboxylic acid group and the pyridinium groups share equal electron-accepting properties. These involvements in fluorescence and the shorter lifetimes may also indicate a new nonradiative deactivation path. On the other hand, the aniline group of **1d** and **2d** causes a drastic quenching of fluorescence in both solvents. Indeed, primary amines (because of their electron lone-pair) are well-known to favor intramolecular electron-transfers (IET), which cause emission quenching [64]. The lifetimes (shorter and bi-exponential for **1d** and not even determined for **2d**) are consistent with this behavior. A drop in pH (by addition of minute amounts of acetic acid, data not shown) induces a partial restoration of **2d** fluorescence emission.

Porphyrins **3** and **4** have similar absorption profiles. Moreover, their fluorescence emission spectra are also similar to a classic porphyrin pattern showing a strong Q (0,0) band (Figure 4B). Families **3** and **4** display close photophysical properties, similar to those of tetraphenyl porphyrin (TPP) [65]. However, fluorescence quantum yields of porphyrins **4** are lower than those of other porphyrins in ethanol. In D_2_O, unlike family **2**, the ratio Q (0,0)/Q (0,1) of family **4** is still higher than that of **1** (Table 4). Whereas the increase in solvent polarity leads to an enhancement in fluorescence emission (twice the yield recorded in ethanol) for **4a** and **4c**, it also causes emission quenching of **4b**. Based on the results obtained with **2b**, this is probably due to the enhanced electron-withdrawing properties of the carboxylic acid group in these conditions. Moreover, the core of porphyrins **4** seems to have a lower pKa than other porphyrins (green coloration of pH = 2 solutions), which could indicate that the phenylimidazole groups behave as EDG. Thus, in D_2_O, in the case of **4b**, a photoinduced intramolecular charge-transfer (ICT) may occur, as this compound is actually behaving as a donor-acceptor (phenylimidazolium groups and carboxylic acid groups, respectively). ICT is well-known to lead to red-shifted and lower fluorescence emissions. In order to verify this hypothesis, an additional porphyrin carrying a primary amine, from this family (**4d**), has been tested and no quenching was observed (Figure S19). As the replacement of the EWG by an EDG involved the recovery of the emissions, our hypothesis seems to be consistent with the data.

Even if **5** and **6** seemed to display similar absorption spectra, some discrepancies have been spotted. First, the Q_x_ (0,0) band is very sensitive to the number of carboxylic functions. Indeed, for family **5**, a slight blue shift (Δλ = 3 nm) of the Q_x_ (0,0) band, as well as a hypochromic effect, and a full-width at half-maximum (fwhm) decrease were observed with **5b**, in comparison with **5a**; the same remarks hold for **5e**, following the increasing number of carboxylic acid groups on the bay region of the porphyrins (Table 4). Regarding porphyrins **6a** and **6b**, Q_x_ (0,0) is also red-shifted (Δλ = 3 nm), but the hypochromic effect is less pronounced. Nonetheless, the intense hypochromic effect and enlargement of the Q_x_ (0,0) band were observed for **6e**. For this compound, moreover, a split of this band was recorded. In the case of **5e**, a split can almost be distinguished. The symmetrization of the porphyrin leads to this effect, in accordance with the four-orbital model of Gouterman [66]. Secondly, a shoulder at 444 nm was recorded in the absorption spectrum of **6a** (Figure 3). This is probably due to a J_2_-aggregation that has already been described for some cationic porphyrins [67]. Whereas the weak shifts in fluorescence emissions of **5** and **6** are consistent with their absorbance, a usual profile with a stronger Q(0,0) band was obtained with **5a**, **5b**, **6a**, and **6b** in ethanol (Figure 4C). When comparing **1** with **2**, imidazole groups seem to have a weaker electron-withdrawing effect, probably because of the steric hindrance (and thus absence of planarity and lack of conjugation), in a manner identical to that of the *ortho*-pyridinium groups [63]. Once again, the carboxylic acid group has a weak involvement on the fluorescence emission in ethanol, whereas the decrease in the ratio Q (0,0)/Q (0,1) of **6b** in D_2_O indicates a stronger electron-withdrawing effect of this group in a polar solvent. On the other hand, **5e** and **6e** have a weak Q (0,0) band, which is consistent with the low epsilon value recorded from their respective Q_x_ (0,0) and Q_y_ (0,0) bands.

### 2.3. Singlet Oxygen Production

Overall singlet oxygen quantum yields of **1** and **2** are significantly high in either ethanol or D_2_O (0.65 < Φ_Δ_ < 0.93) (Table 4). The presence of one carboxylic acid group seems to slightly increase the different yields, even if **2a** has a lower yield in ethanol, probably because of its aggregation. However, **1d** and **2d** were characterized by a very low singlet oxygen production (not even measurable), because of, once again, the quenching of emissions induced by the IET. Nevertheless, after the addition of a few drops of acetic acid (data not shown), a characteristic singlet oxygen emission was observed for **2d**. According to the literature, the presence of bacteria may also induce a restoration of the production of singlet oxygen, as this PS has shown a photobactericidal activity [73]. As previously mentioned, families **3** and **4** have shown results similar to those of TPP. Indeed, the singlet oxygen production yield of these porphyrins in ethanol is ca. 0.55 (0.49 < Φ_Δ_ < 0.66), which is very similar to the TPP–COOH value (0.54). However, in D_2_O, porphyrins **4a** and **4c** have shown a significant singlet oxygen production increase (Φ_Δ_ = 0.83 and 0.62, respectively). Efficient singlet oxygen productions have also been observed with porphyrins **5** and **6** (0.58 < Φ_Δ_ < 0.86) in ethanol. In D_2_O, these values are, once again, slightly higher. In both families **5** and **6**, in ethanol as in D_2_O, the singlet oxygen production quantum yield increases with the number of carboxylic groups and with the symmetrization of the porphyrin (Φ_Δ_ = 0.86 for **6e** in D_2_O).

Based on this study, different phenomena have been observed in the various families of porphyrins. Whereas intramolecular transfers (especially in aqueous solutions) have been detected in pyridyl and phenylimidazole-substituted porphyrins and their *N*-methyl derivatives, methylimidazole groups induce fewer effects on the porphyrin core. Moreover, the symmetrization of imidazole porphyrins has a positive influence on their singlet oxygen production. Thus, studies based on asymmetric cationic porphyrins should include a judicious selection of the kind of porphyrin in order to avoid any quenching in aqueous solution (especially if they are supposed to serve as controls). The presence of one carboxylic acid group in **1, 2, 5,** and **6** seems to slightly increase the production of singlet oxygen, whereas a carboxylic acid group should be avoided in **4**. On the other hand, methylimidazolium porphyrins **6** seem to be promising candidates for the studies that plan to use two coupled moieties in *trans* positions, as **6e** has shown a significant singlet oxygen production in aqueous solution.

### 2.4. Bacterial Photoinactivation

We compared the bacterial photoinactivation efficiency of each porphyrin family against three bacterial species known to be involved in nosocomial infections (*Staphylococcus aureus*, *Escherichia coli*, and *Pseudomonas aeruginosa*). This antibacterial study focused on *N*-methylporphyrins. Indeed, pyridinium porphyrin **2a** is already renowned as a better PACT photosensitizer than **1a** [29], which may suggest a similar difference between other porphyrins and their respective *N*-methyl form. As the functionalized porphyrins (**2b**, **2c**, **4b**, **4c**, **6b**, **6e**) have been devised in view of their further optimized derivatization and use in PACT, only A_4_ porphyrins **2a**, **4a**, and **6a** were used in these biological assays. Moreover, the impact due to the different cationic groups can be better compared in the absence of any interfering chemical group.

The photoinactivation capacity of compounds **2a**, **4a**, and **6a** has been confirmed (Table 5). Inactive in the dark, these porphyrins led to bacterial inhibition of all strains after light irradiation. Despite the narrow range of the minimum bactericidal concentrations (MBCs), porphyrin **4a** was more efficient against *S. aureus* (1.5 μM). Assays against *E. coli* revealed discrepancies between the different PS. Whereas **4a** was characterized by a low MBC (2.5 μM), significantly higher concentrations of **2a** and **6a** were required to photoinactivate this strain (>18.0 μM). Concerning *P. aeruginosa*, all MBCs tightly clustered around 22.0 μM. In comparison with porphyrin **2a** (already known as an efficient PS [74]), **6a** showed the highest MBCs against all strains. Based on the presumptive relation between PS efficacy and their positive charge, this result is surprising as this porphyrin bears the same positive charge, as **2a** and **4a**; this positive charge is distributed on substituents of smaller size that then present a higher positive charge density. Thus, this lower activity is probably due to the lower production of singlet oxygen of **6a**. Porphyrin **4a** has shown promising results against *S. aureus* and, interestingly, against *E. coli*. Considering MBCs, the efficiency of **4a** can be estimated as 7 times better than that of **2a** against *E. coli*. The photophysical properties and, especially, the respective singlet oxygen productions of **2a** and **4a** do not offer enough discrepancy to justify these results. Thus, a specific interaction between **4a** and *E. coli* could be the reason of this higher efficiency.

**Figure 5:**
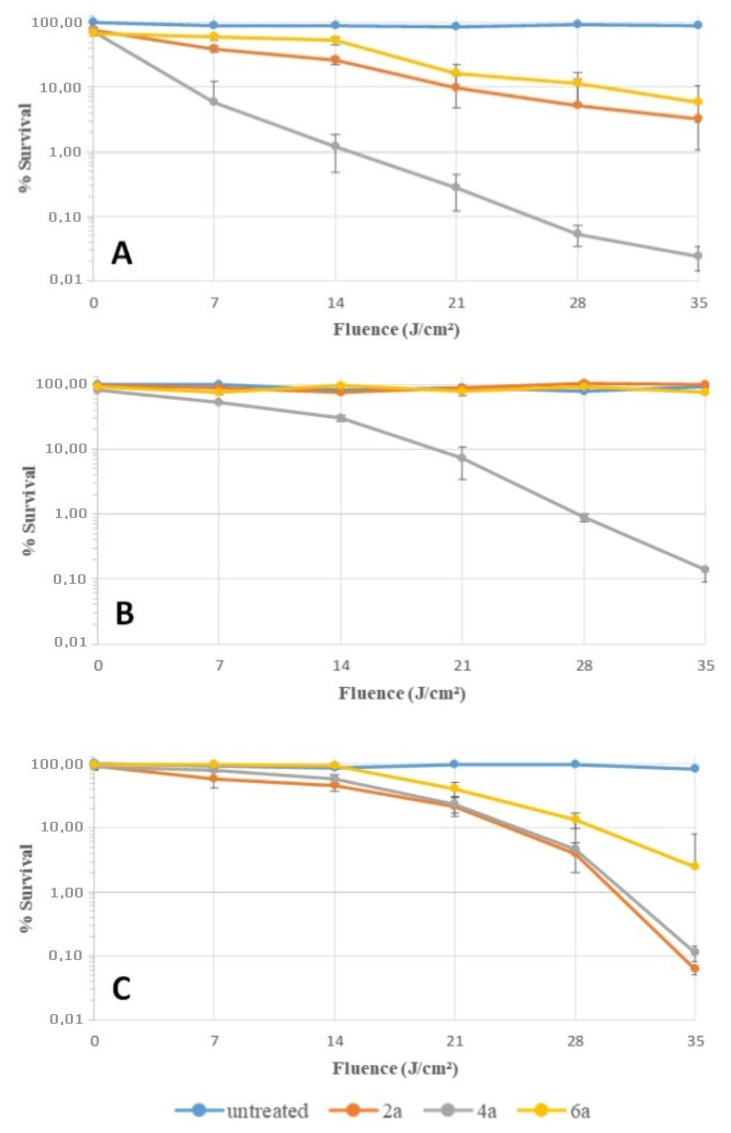
Time course of cell survival during photodynamic inactivation of *S. aureus* (**A**), *E. coli* (**B**), and *P. aeruginosa* (**C**). Bacteria were incubated for 30 min in the dark at 37 °C in solutions of porphyrins **2a**, **4a**, and **2a** (2 μM for *S. aureus* and *E. coli*, 20 μM for *P. aeruginosa*). After 2 washings with PBS, bacteria were irradiated by white LEDs (4.83 mW/cm²). Survival rates were expressed as percent of the initial counts and plotted as a function of cumulative light fluence (J/cm²).

An additional experiment was performed to confirm these previous results (Figure 5). Bacteria were harvested and put in contact with the different compounds in PBS. After 30 min of incubation at 37 °C in the dark, bacteria were washed twice, resuspended in PBS, and then irradiated as before. The steepest decline in survival rate was obtained with *S. aureus* irradiated after the contact with porphyrin **4a**. Indeed, a fluence of 14 J/cm² induced a reduction of 2 log in the survival rate; compounds **2a** and **6a** were signficantly less efficient. This result could be due to a better affinity of **4a** for the cell envelope of *S. aureus*. Interestingly, a fluence of 35 J/cm² with this same compound induced a reduction of 3 log in the survival rate of *E. coli* in accordance with MBC values (Table 5). For *P. aeruginosa*, a fluence of 35 J/cm² was also necessary to induce a reduction of 3 log in the survival rate, but in presence of a higher concentration of the cationic porphyrins during the incubation (2 µM for *E. coli* against 20 µM for *P. aeruginosa*). This set of assays showed that porphyin **4a** has a better affinity for bacteria, especially for *S. aureus* and *E. coli*, than others porphyrins. This effect allows this compound to induce a better photocidal activity against these strains, which is coherent with the first set of biological assays.

## 3. Materials and Methods

### 3.1. General Methods

All organic materials and microbial nutrients were purchased from commercial suppliers (Acros Organics, Alfa Aesar, Grosseron, Sigma-Aldrich, TCI). All reactants for the synthesis of porphyrins were obtained from Sigma-Aldrich and directly used, except ethyl 4-formylphenoxyacetate, which was prepared from 4-hydroxybenzaldehyde. Solvents used for the UV-Vis and fluorescence measurements were of spectroscopic grade and were stored in a dark place. Analytical thin-layer chromatography (TLC) was performed on Merck 60F254 silica gel. Column chromatography was carried out with silica gel (60 ACC, 15–40 μm, Merck, Darmstadt, Germany). Reverse-phase chromatography was performed on a RediSep^®^Rf C18 column (43 g) mounted on an Interchim^®^ puriFlash™ 430 apparatus (provided by Interchim, Montluçon, France) with acetonitrile (HPLC grade +0.1% TFA) and distilled water (+0.1%TFA) as eluents. Continuous monitoring of effluent absorbance allowed the detection of cationic porphyrins (λ = 418 nm). ^1^H and ^13^C nuclear magnetic resonance (^1^H-NMR and ^13^C-NMR) spectra were recorded in deuterated solvents, with Bruker DPX 400 and 500 spectrometers. Chemical shifts are reported as δ (parts per million), downfield from internal TMS. Multiplicities are reported as follows: s = singlet, d = doublet, m = multiplet. Electron spray ionization mass spectra (ESI-MS) were recorded on a Sciex 4000 Q-TRAP^®^, monitored by Sciex Analyst 1.6.2 (Applied Biosystems, Loughborough, UK).

### 3.2. Chemical Synthesis

#### 3.2.1. Preparation of Ethyl 4-Formylphenoxyacetate

4-hydroxybenzaldehyde (1.00 g, 8.20 mmol, 1 eq), ethyl bromoacetate (0.90 mL, 8.20 mmol, 1 eq), and a large excess of K_2_CO_3_ (3 g) were stirred in a solution of DMF (100 mL) for 3 h at 95 °C. The reaction was monitored by TLC (hexane/ethyl acetate (70/30, *v*/*v*)). After removal of the solvent in vacuo, the product was extracted in CHCl_3_, washed with distilled water, and dried over MgSO_4_. The solvent was removed and the protected aldehyde was obtained as a yellowish powder (1.53 g, yield 90%).

#### 3.2.2. General Procedure for the Synthesis of Porphyrins

Freshly distilled pyrrole (6.66 mL, 96.0 mmol, 4 eq) was added dropwise to a refluxed solution of propionic acid (120 mL) containing aromatic aldehyde (4-pyridylcarboxaldehyde, 4-imidazolebenzaldehyde, or *N*-methyl-4-imidazolecarboxaldehyde, 4 eq for A_4_ porphyrins, 3 eq for A_3_B porphyrins, 2 eq for A_2_B_2_ porphyrins) and functionalized aldehyde (methyl 4-formylbenzoate, 4-acetamidobenzaldehyde, or ethyl 4-formylphenoxyacetate, 1 eq for A_3_B porphyrins, 2 eq for A_2_B_2_ porphyrins). The mixture was stirred under reflux for 3 h. After removal of propionic acid in vacuo, the crude solid was dissolved in CHCl_3_, filtered through a small pad of silica gel, and washed with CHCl_3_/EtOH (90/10, *v*/*v*). After removal of the solvent under vacuum, porphyrins were purified by silica gel column chromatography (CHCl_3_/EtOH (98/2, *v*/*v*)) to afford A_4_ porphyrin (yield 10% for **1a**, yield 15% for **3a**, yield 2% for **5a**), A_3_B porphyrin (yield 2–5% for **1**, yield 10–13% for **3**, yield 5% for **5**), or A_2_B_2_ porphyrin (yield 5% for **5**) as light purple powders.

**1a (tetrakis(4-pyridyl)porphyrin):**^1^H NMR (500 MHz, CDCl_3_): δ (ppm) = 9.06 (d, *J* = 6.0 Hz, 8H, H*o*-py), 8.87 (s, 8H, Hpyrr), 8.16 (d, *J* = 6.0 Hz, 8H, H*m*-py), -2.92 (s, 2H, NH); MS (ESI+) [C_40_H_26_N_8_]: calcd [M + 2H]^2+^ 310.11, found 310.31.

**3a (tetrakis(4-imidazol-phenyl)porphyrin):**^1^H NMR (500 MHz, CDCl_3_ 9/1 CD_3_OD): δ (ppm) = 8.92 (s, 8H, Hpyrr), 8.36 (d, *J* = 8.3 Hz, 8H, H*o*-phe), 8.26 (s, 4H, Him-2), 7.86 (d, *J* = 8.3 Hz, 8H, H*m*-phe), 7.67 (s, 4H, Him-5), 7.34 (s, 4H, Him-4); MS (ESI+) [C_56_H_38_N_12_]: calcd [M + 2H]^2+^ 440.16, found 440.04.

**5a (tetrakis(*N*-methylimidazol-2-yl)porphyrin):**^1^H NMR (500 MHz, CDCl_3_ 9/1 CD_3_OD): δ (ppm) = 8.88 (m, 8H, Hpyrr), 7.67 (d, *J* = 1.3 Hz, 2H, Him-5), 7.65 (d, *J* = 1.3 Hz, 2H, Him-4), 7.51 (d, *J* = 1.1 Hz, 2H, Him-5), 7.50 (d, *J* = 1.1 Hz, 2H, Him-4), 3.45 (m, 12H, N-_CH3_); MS (ESI+) [C_36_H_30_N_12_]: calcd [M + 2H]^2+^ 316.13, found 316.26.

#### 3.2.3. General Procedure for the Deprotection of Carboxylic Acid

Esters of A_3_B porphyrins (200 µmol) were saponified in a refluxing solution of KOH (2 mL, 2 mol/L) and EtOH (for methyl ester) or MeOH (for ethyl ester) (50 mL) for 24 h. After removal of the solvent, an excess of HCl was added until the green coloration of the solution appeared. The crude product was extracted in CHCl_3_/TEA (99/1, *v*/*v*), washed with distilled water, and dried over MgSO_4_. The residue was purified by silica gel column chromatography (CHCl_3_/EtOH (80/20, *v*/*v*)) to obtain A_3_B porphyrins with deprotected carboxylic acid functions, as purple solids.

**1b (5-(4-carboxyphenyl)-10,15,20-tri(4-pyridyl)-*21H*,*23H*-porphyrin):** 178 µmol, yield 89%. ^1^H NMR (500 MHz, CDCl_3_ 9/1 CD_3_OD): δ (ppm) = 9.40 (d, *J* = 6.5 Hz, 6H, H*o*-py), 8.97 (m, 6H, Hpyrr), 8.93 (t, *J* = 6.5 Hz, 6H, H*m*-py), 8.87 (d, *J* = 4.7 Hz, 2H, Hpyrr-phe), 8.58 (d, *J* = 8.0 Hz, 2H, H*o*-phe), 8.42 (d, *J* = 8.0 Hz, 2H, H*m*-phe); MS (ESI+) [C_42_H_27_N_7_O_2_]: calcd [M + 2H]^2+^ 331.61, found 331.64.

**3b (5-(4-carboxyphenyl)-10,15,20-tri(4-imidazol-phenyl)-*21H*,*23H*-porphyrin):** 182 µmol, yield 91%. ^1^H NMR (500 MHz, CD_2_Cl_2_ 9/1 CD_3_OD): δ (ppm) = 8.83 (s, 8H, Hpyrr), 8.35 (d, *J* = 8.2 Hz, 2H, H*o*-phe_COOH_), 8.23 (m, 11 H, H*m*-phe_COOH_ + H*o*-phe + Him-2), 7.77 (d, *J* = 8.3 Hz, 6H, H*m*-phe), 7.62 (s, 3H, Him-5), 7.24 (s, 3H, Him-4); MS (ESI+) [C_54_H_36_N_10_O_2_]: calcd [M + 2H]^2+^ 429.15, found 429.03.

**3c (5-(4-phenyoxyacetic)-10,15,20-tri(4-imidazol-phenyl)-*21H*,*23H*-porphyrin):** 184 µmol, yield 92%. ^1^H NMR (500 MHz, CDCl_3_ 9/1 CD_3_OD): δ (ppm) = 8.88 (m, 8H, Hpyrr), 8.31 (d, *J* = 8.1 Hz, 6H, H*o*-phe), 8.19 (s, 3H, Him-2), 8.14 (d, *J* = 8.5 Hz, 2H, H*o*-phe_o_), 7.78 (d, *J* = 8.1 Hz, 6H, H*m*-phe), 7.59 (s, 3H, Him-5), 7.38 (s, 3H, Him-4), 7.31 (d, *J* = 8.5 Hz, 2H, H*m*-phe_o_), 4.77 (s, 2H, O-CH_2_-), −2.75 (s, 2H, NH); MS (ESI+) [C_55_H_38_N_10_O_3_]: calcd [M +2H ]^2+^ 444.15, found 444.17.

**5b (5-(4-carboxyphenyl)-10,15,20-tri(*N*-methylimidazol-2-yl)-*21H*,*23H*-porphyrin):** 172 µmol, yield 86%. ^1^H NMR (500 MHz, CDCl_3_ 9/1 CD_3_OD): δ (ppm) = 8.88 (m, 8H, Hpyrr), 8.42 (m, 2H, H*o*-phe_COOH_), 8.19 (m, 2H, H*m*-phe_COOH_), 7.65 (m, 3H, Him-5), 7.51 (m, 3H, Him-4), 3.45 (m, 9H, N–CH_3_); MS (ESI+) [C_39_H_30_N_10_O_2_]: calcd [M +2H]^2+^ 336.13, found 336.15.

**5e (di(4-carboxyphenyl)- di(*N*-methylimidazol-2-yl)-*21H*,*23H*-porphyrin):** 174 µmol, yield 87%. ^1^H NMR (500 MHz, CDCl_3_): δ (ppm) = 8.85 (m, 8H, Hpyrr), 8.43 (m, 4H, H*o*-phe_COOH_), 8.19 (m, 4H, H*m*-phe_COOH_), 7.67 (s, 2H, Him-5), 7.47 (s, 2H, Him-4), 3.44 (m, 6H, N–CH_3_), −2.78 (m, 2H, NH); MS (ESI+) [C_42_H_30_N_8_O_4_]: calcd [M + 2H]^2+^ 356.12, found 356.16.

#### 3.2.4. General Procedure for the *N*-Methylation of Porphyrins

An excess of iodomethane (60 eq for A_4_ porphyrins, 45 eq for A_3_B porphyrins, 30 eq for A_2_B_2_ porphyrins) was added to a solution of porphyrin (1 eq) dissolved in anhydrous DMF (25 mL). The mixture was stirred for 3 h at 140 °C. DMF and excess iodomethane were removed with a rotary vane pump under heating (70 °C) to obtain *N*-methyl porphyrins in quantitative yields as light purple powders without further purification.

**2a (tetrakis(4-*N*-methylpyridyl)porphyrin):**^1^H NMR (500 MHz, DMSO_d6_): δ (ppm) = 9.49 (d, *J* = 6.0 Hz, 8H, H*o*-py), 9.17(s, 8H, Hpyrr), 8.99 (d, *J* = 6.0 Hz, 8H, H*m*-py), 4.74 (s, 12H + water residual, N^+^–CH_3_), −3.09 (s, 2H, NH); MS (ESI+) [C_44_H_38_I_4_N_8_]: calcd [M + H]^4+^ 169.83, found 169.81.

**2b (5-(4-carboxyphenyl)-10,15,20-tri(4-*N*-methylpyridyl)-*21H*,*23H*-porphyrin):**^1^H NMR (500 MHz, DMSO_d6_): δ (ppm) = 9.49 (d, *J* = 6.1 Hz, 6H, H*o*-py), 9.15 (m, 4H, Hpyrr), 9.07 (m, 4H, Hpyrr-phe), 9.01 (d, *J* = 6.2 Hz, 6H, H*m*-py), 8.41 (m, 4H + chloroform residual, Hphe), 4.73 (s, 9H, N^+^–CH_3_), −2.99 (s, 2H, NH); MS (ESI+) [C_45_H_36_I3N_7_O_2_]: calcd [M]^2+^ 353.14, found 352.65.

**4a (tetrakis(4-*N*-methylimidazol-phenyl)porphyrin):**^1^H NMR (500 MHz, DMSO_d6_): δ (ppm) = 10.13 (s, 4H, Him-2), 8.93 (s, 8H, Hpyrr), 8.65 (s, 4H, Him-4), 8.53 (d, *J* = 8.3 Hz, 8H, H*o*-phe), 8.27 (d, *J* = 8.3 Hz, 8H, H*m*-phe), 8.14 (s, 4H, Him-5), 4.10 (s, 12H, N^+^–CH_3_), −2.88 (s, 2H, NH); MS (ESI+) [C_60_H_50_I_4_N_12_]: calcd [M]^4+^ 234.60, found 234.70.

**4b (5-(4-carboxyphenyl)-10,15,20-tri(4-*N*-methylimidazol-phenyl)-*21H*,*23H*-porphyrin):**^1^H NMR (500 MHz, DMSO_d6_): δ (ppm) = 10.13 (s, 3H, Him-2), 8.93 (s, 6H, Hpyrr), 8.89 (s, 2H, *J* = 4.2 Hz, Hpyrr-phe_COOH_), 8.65 (s, 3H, Him-4), 8.54 (d, *J* = 8.2 Hz, 6H, H*o*-phe), 8.42 (d, *J* = 8.1 Hz, 2H, H*o*-phe_COOH_), 8.36 (d, *J* = 8.1 Hz, 2H, H*m*-phe_COOH_), 8.26 (d, *J* = 8.3 Hz, 6H, H*m*-phe), 8.14 (s, 3H, Him-4), 4.10 (s, 9H, N^+^–CH_3_), −2.88 (s, 2H, NH); MS (ESI+) [C_57_H_45_I_3_N_10_O_2_]: calcd [M]^3+^ 300.47, found 300.41.

**4c (5-(4-phenyoxyacetic)-10,15,20-tri(4-*N*-methylimidazol-phenyl)-*21H*,*23H*-porphyrin):**^1^H NMR (500 MHz, DMSO-*d*_6_): δ (ppm) = 10.13 (s, 3H, Him-2), 8.96 (d, *J* = 4.1 Hz, 2H, Hpyrr), 8.92 (s, 4H, Hpyrr), 8.88 (d, *J* = 4.1 Hz, 2H, Hpyrr), 8.65 (s, 3H, Him-4), 8.54 (d, *J* = 8.3 Hz, 6H, H*o*-phe) 8.26 (d, *J* = 8.3 Hz, 6H, H*m*-phe), 8.15 (d, *J* = 8.6 Hz, 2H, H*o*-phe_o_ & s, 3H, Him-5), 7.40 (d, *J* = 8.6 Hz, 2H, H*m*-phe), 4.99 (s, 2H, O–CH_2−_), 4.10 (s, 9H, N^+^–CH_3_), −2.87 (s, 2H, NH); MS (ESI+) [C_58_H_47_I_3_N_10_O_3_]: calcd [M + H]^3+^ 310.79, found 310.75.

**6a (tetrakis(*N,N’*-dimethylimidazol-2-yl)porphyrin):**^1^H NMR (500 MHz, DMSO_d6_): δ (ppm) = 9.40 (s, 8H, Hpyrr), 8.55 (s, 8H, Him), 3.75 (s, 24H, N–CH_3_), −3.23 (s, 2H, NH); MS (ESI+) [C_40_H_42_I_4_N_12_]: calcd [M]^4+^ 172.59, found 172.54.

**6b (5-(4-carboxyphenyl)-10,15,20-tri(*N,N’*-dimethylimidazol-2-yl)-*21H*,*23H*-porphyrin):**^1^H NMR (500 MHz, DMSO_d6_): δ (ppm) = 9.23 (m, 8H, Hpyrr), 8.52 (s, 6H, Him), 8.47 (d, *J* = 8.2 Hz, 2H, H*o*-phe_COOH_), 8.39 (d, *J* = 8.2 Hz, 2H, H*m*-phe_COOH_), 3.75 (m, 18H, N–CH_3_), −3.03 (s, 2H, NH); MS (ESI+) [C_42_H_39_I_3_N_10_O_2_]: calcd [M]^3+^ 238.44, found 238.50.

**6e (di(4-carboxyphenyl) -di(*N,N’*-dimethylimidazol-2-yl)-*21H*,*23H*-porphyrin):**^1^H NMR (500 MHz, DMSO_d6_): δ (ppm) = 9.24–8.95 (m, 8H, Hpyrr), 8.51 (s, 4H, Him), 8.43 (d, *J* = 8.1 Hz, 4H, H*o*-phe_COOH_), 8.38 (d, *J* = 8.1 Hz, 2H, H*m*-phe_COOH_), 3.75 (s, 12H, N-CH_3_), −2.81 (s, 2H, NH); MS (ESI+) [C_44_H_36_I_2_N_8_O_4_]: calcd [M]^2+^ 370.14, found 370.04.

#### 3.2.5. Synthesis of 5-(4-minophenyl)-10,15,20-tri(4-pyridyl)-21H,23H-porphyrin (**1d**)

5-(4-acetamidophenyl)-*10,15,20*-tri(4-pyridyl)-2*1H,23H*-porphyrin (250 mg, 0.373 mmol) was dissolved into a solution of HCl (5 M, 250 mL) under reflux for 3 h. After removal of the solvent in vacuo, the product was extracted in CHCl_3_/TEA (90/10, *v*/*v*), washed with distilled water, and dried over MgSO_4_. The solvent was removed to afford **1d** as a purple powder with a quantitative yield.

**1d:**^1^H NMR (500 MHz, CDCl_3_): δ (ppm) = 9.02 (d, *J* = 5.5 Hz, 6H, H*o*-py), 9.02 (d, *J* = 2.5 Hz, 2H, Hpyrr-phe), 8.81 (m, 6H, Hpyrr), 8.16 (d, *J* = 5.5 Hz, 6H, H*m*-py), 7.98 (d, *J* = 8.0 Hz, 2H, H*o*-phe), 7.08 (d, *J* = 8.0 Hz, 2H, H*m*-phe); MS (ESI+) [C_41_H_28_N_8_]: calcd [M + 2H]^2+^ 317.12, found 316.93.

#### 3.2.6. Synthesis of 5-(4-aminophenyl)-10,15,20-tri(4-*N*-methylpyridyl)-*21H,23H*-porphyrin (**2d**)

5-(4-acetamidophenyl)-*10,15,20*-tri(4-pyridyl)-*21H,23H*-porphyrin was directly *N*-methylated, following the previous described procedure. Then, the crude was diluted in a solution of H_2_O/TFA (75/25, *v*/*v*) and stirred under reflux for 5 h. The aqueous solution was removed and the product was freeze-dried to obtain **2d** as a purple-brown powder with a quantitative yield.

**2d:**^1^H NMR (500 MHz, DMSO_d6_): δ (ppm) = 9.49 (d, *J* = 6.5 Hz, 6H, H*o*-py), 9.11 (m, 8H, Hpyrr), 9.01 (m, 2H, Hpyrr-phe), 9.00 (d, *J* = 6.5 Hz, 4H, H*m*-py), 8.97 (d, *J* = 6.5 Hz, 2H, H*m*-py), 7.94 (d, *J* = 8.5 Hz, 2H, H*o*-phe), 7.15 (d, *J* = 8.5 Hz, 2H, H*m*-phe), 4.73 (s, 9H, N^+^–CH_3_), −2.96 (s, 2H, NH); MS (ESI+) [C_44_H_37_I_3_N_8_]: calcd [M]^3+^ 225.77, found 225.74.

### 3.3. Spectroscopic Measurements

Absorption spectra were recorded on a UV-3600 UV-visible double-beam spectrophotometer (Shimadzu, Marne La Vallée, France). Fluorescence spectra were recorded on a Fluorolog FL3-222 spectrofluorometer (Horiba Jobin Yvon, Longjumeau, France) equipped with a 450 W Xenon lamp, a thermostated cell compartment (25 °C), a UV-visible R928 photomultiplier (Hamamatsu, Japan), and an InGaAs infrared detector (DSS-16A020L Electro-Optical System Inc, Phoenixville, PA, USA). The excitation beam was diffracted by a double-ruled-grating SPEX monochromator (1200 grooves/mm blazed at 330 nm). The emission beam was diffracted by a double-ruled-grating SPEX monochromator (1200 grooves/mm blazed at 500 nm). Singlet oxygen emission was detected through a double-ruled-grating SPEX monochromator (600 grooves/mm blazed at 1 μm) and a long-wave pass (780 nm). All spectra were recorded from solutions introduced in four-face quartz cuvettes. All the emission spectra (fluorescence and singlet oxygen luminescence) have been displayed with the same absorbance (less than 0.2) with lamp and photomultiplier correction. Fluorescence quantum yields (Φ_F_) were determined using a tetraphenyl porphyrin (TPP) solution in toluene as the fluorescence standard (Φ_F_ = 0.11). The quantum yield of ^1^O_2_ production was determined by direct analysis of the ^1^O_2_ near-infrared luminescence at 1270 nm. Whereas TPP–COOH was used as standard in ethanol (Φ**_Δ_** = 0.54), tetra(*N*-methylpyridyl)porphyrin (**2a**) was chosen as the reference solution in D_2_O due to its high ^1^O_2_ quantum yield (Φ_Δ_ = 0.90). Time-resolved experiments were performed using for excitation a pulsed laser diode emitting at 407 nm (LDH-P-C-400M, FWHM < 70 ps, 1 MHz) coupled with a PDL 800-D driver (both from PicoQuant GmbH, Berlin, Germany), and for detection from an avalanche photodiode SPCM-AQR-15 (EG and G, Vaudreuil, QC, Canada) coupled with a 550 nm long-wave pass filter as the detection system. Acquisition was performed by a PicoHarp 300 module with a PHR-800 4-channel router (both PicoQuant GmbH, Berlin, Germany). Fluorescence decays were recorded using the single photon counting method. Data were collected up to 1000 counts accumulated in the maximum channel and analyzed using time-correlated single-photon counting (TCSPC) software (PicoQuant GmbH, Berlin, Germany) based on iterative reconvolution using a Levensberg–Marquandt algorithm, enabling the obtention of multi-exponential profiles (mainly one or two exponentials in our cases). Singlet oxygen lifetime measurements were performed on a TEMPRO-01 spectrophotometer (Horiba Jobin Yvon, Longjumeau, France) composed of a SpectraLED-415 pulsed diode excitation source emitting at 415 nm, a cell compartment, a Seya-Namioka-type emission monochromator (600–2000 nm), and a H10330-45 near-infrared photomultiplier tube with a thermoelectric cooler (Hamamatsu, Japan) as the detection system. The system was monitored by a FluoroHub-B single-photon counting controller and the DataStation and DAS6 softwares (Horiba Jobin Yvon). Emission spectra were recorded on a Fluorolog FL3-22 spectrofluorometer (Horiba Jobin Yvon, Longjumeau, France) equipped with a 450 W Xenon lamp, a xenon flash lamp, a thermostated cell compartment (25 °C), and a R928 UV-visible photomultiplier (Hamamatsu, Japan). The system was monitored by a FluoroHub-B single-photon counting controller. The FluorEssence™ software (Horiba Jobin Yvon) was used for recording emission spectra. The DataStation and DAS6 softwares (Horiba Jobin Yvon) were used for phosphorescence lifetime measurements.

### 3.4. Bacterial Cultures

Gram-positive (*S. aureus* CIP76.25) and Gram-negative (*P. aeruginosa* CIP76110 and *E. coli* CIP54.8T) bacterial strains were obtained from Institut Pasteur (Paris, France). These strains were cultured in liquid tryptic soy (pancreatic casein extract 17 g/L, soy flour papaic digest 3 g/L, dextrose 2.5 g/L, NaCl 5 g/L, and K_2_HPO_4_ 2.5 g/L) and incubated overnight at 37 °C under aerobic conditions. Square plates were kept warm inside a SR1000 Thermosi incubator. Bacterial concentrations were estimated by reading the turbidity at 600 nm in a JENWAY 6320D spectrophotometer (Cole-Parmer Ltd., Roissy, France). Antimicrobial experiments used a Heidolph Unkubator 1000 equipped with an Unimax 1010 orbital platform shaker (Heidolph Instruments GmbH & CO. KG, Schwabach, Germany). An Isotech Lightmeter 1335 light meter was used to measure the effective irradiation power (RS Components, CorbyNorthants, UK).

### 3.5. Bacterial Photoinactivation

A_4_ porphyrins were added to bacterial cultures during the log phase. However, **1a**, **3a**, and **5a** have poor solubility in DMSO and in aqueous solution. The bacterial assays revealed that these compounds aggregated in the culture broth and precipitated during incubation, which prevented any further observation (data not shown). Fresh solutions of porphyrin **2a**, **4a**, and **6a** in DMSO were mixed with tryptic soy culture medium. The DMSO concentration never exceeded 1% (*v*/*v*). From these mixtures, 1 mL aliquots of serial dilutions (100 μM down to 156 nM) were transferred into two 24-well plates (BD Falcon). Then, 1 mL of cultures containing 2 × 10^5^ CFU/mL were deposited in each well. The 24-well plates were irradiated with LED visible light. The fluence rate (4.83 mW/cm²) was measured with a light meter. Plates were incubated under shaking (200 rpm) at 37 °C for 20 h (totaling 348 J/cm² fluence). Controls consisting of 24-well plates were prepared in the same conditions but kept in the dark. Six independent experiments were performed with each strain. The bacterial count was performed after a 10-fold serial dilution of each well. Each dilution was spread on tryptic soy agar plates using an automatic plater (easySPIRAL^®^, Interscience, Saint-Nom-la-Bretêche, France). After incubation at 37 °C for 24 h, colonies were counted to determine the total CFU per mL (CFU/mL). The minimum bactericidal concentration (MBC) corresponds to the concentration of the active compound for which 99.9% of the bacteria have been killed (i.e., 3 log reduction compared to the untreated control).

To observe the effects of light fluence (J/cm²) on the survival rate of each strain, bacteria (~10^9^ CFU/mL) were incubated for 30 min at 37 °C in the dark with 1 mL of the different solutions (2 μM for *S. aureus* and *E. coli*, 20 μM for *P. aeruginosa*). Then, bacteria were collected by centrifugation (10,000 rpm, 5 min) and washed twice with PBS 1 × (without Ca^2+^ and Mg^2+^). Washed bacteria were resuspended in PBS, transferred into a 96-well plate, and irradiated by white LEDs (4.83 mW/cm²). Survival rates, calculated in relation to the initial count of each bacterial culture, were plotted as a function of cumulative fluence (J/cm²). Four independent experiments were performed with each strain.

## 4. Conclusions

In order to photoinactivate MDR strains, previous studies have highlighted the efficiency of cationic PS, including pyridinium porphyrins. In this work, different families of cationic porphyrin have been investigated to support future studies. Even if pyridinium A_4_ porphyrins are still very efficient, the functionalization of this family may induce some reactivity issues (particularly in the case of the carboxylic acid function) or emissions quenching (due to the presence of primary amine functions). Even if the participation of a carboxylic acid function leads to emission quenching of these PS (as a consequence of an ICT), phenylimidazolium porphyrins have shown several advantages, as easier synthesis, consistent photophysical properties (similar to those of TPP), along with a significantly high photoinactivation activity against *S. aureus* and *E. coli*. Finally, the methylimidazolium groups have not a strong electron-withdrawing effect on the porphyrin core, which allows the designing of cationic PSs with different chemical functions without any emissions quenching due to potential ICT/IET. Whereas **6a** showed less efficiency against bacteria, likely due to lower oxygen singlet production, porphyrin **6e** has a significant singlet oxygen production. Thus, further photobactericidal studies on porphyrins **6** should focus on compounds bearing two targeting groups.

## Figures and Tables

**Table 1:** Structures of neutral and cationic synthetized porphyrins.

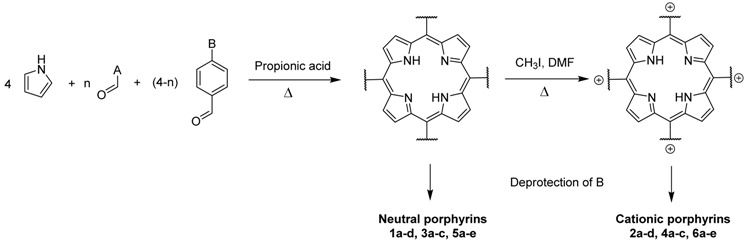					
**Neutral Porphyrins**				**Cationic Porphyrins**	
**n**	**A**	**B**	**Compound ^a^**	**Methylated A**	**Compound ^a^**
4	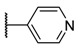	-	**1a** [47,48]	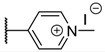	**2a** [47,48]
3	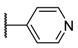	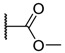	**1b** [49,50]	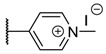	**2b** [51,52]
3	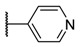	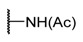	**1d** [53]	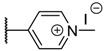	**2d** [44]
4	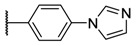	-	**3a** [54,55]	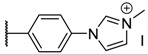	**4a** [32]
3	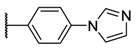	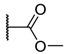	**3b**	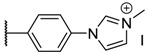	**4b**
3	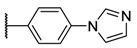	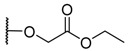	**3c**	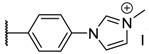	**4c**
4	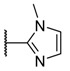	-	**5a** [30]	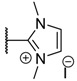	**6a** [30,56]
3	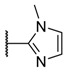	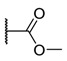	**5b**	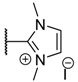	**6b**
2	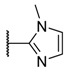	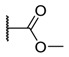	**5e (isomer mixture)** [57]	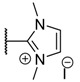	**6e (isomer mixture)**

^a^ Related compounds obtained after deprotection of carboxylic acid or amine functions, followed by previous studies using similar compounds.

**Table 2:** Average yields of porphyrin syntheses.

Type	Porphyrin	Average Yield (%)
A_4_	**1a**	10
	**3a**	15
	**5a**	2
A_3_B	**1b**	5
	**1d**	2
	**3b**	13
	**3c**	10
	**5b**	5
A_2_B_2_	**5e**	5

**Table 3:** Maximum absorbance wavelengths (λ, nm) and molar absorptivities (log ε, L mol^−1^ cm^−1^) of the cationic porphyrins in ethanol solution.

λmax (nm) (log ε; L mol^−1^ cm^−1^)											
Compd	Soret	Qy(1,0)	Qy(0,0)	Qx(1,0)	Qx(0,0)	Compd	Soret	Qy(1,0)	Qy(0,0)	Qx(1,0)	Qx(0,0)
**1a**	414 (3.92)	509 (2.56)	546 (2.00)	588 (2.00)	645 (1.75)	**4a**	416 (5.62)	513 (4.24)	547 (3.86)	590 (3.71)	645 (3.45)
**1b**	415 (5.10)	512 (3.86)	544 (3.51)	589 (3.44)	645 (3.15)	**4b**	417 (5.67)	513 (4.30)	547 (3.94)	590 (3.78)	646 (3.57)
**1d**	416 (4.82)	519 (3.93)	555 (3.78)	594 (3.67)	655 (3.54)	**4c**	418 (5.57)	514 (4.20)	549 (3.89)	591 (3.69)	649 (3.55)
**2a**	415 (5.16)	524 (4.44)	556 (4.26)	594 (4.19)	646 (3.87)	**5a**	416 (5.44)	509 (4.24)	543 (3.48)	585 (3.75)	658 (3.63)
**2b**	426 (5.40)	517 (4.26)	554 (3.91)	592 (3.82)	649 (3.43)	**5b**	415 (5.28)	510 (4.04)	543 (3.36)	586 (3.54)	655 (3.20)
**2d**	428 (5.16)	520 (4.10)	566 (3.93)	593 (3.92)	657 (3.54)	**5e**	415 (5.45)	511 (4.12)	545 (3.52)	586 (3.62)	650 (3.01)
**3a**	417 (3.71)	516 (2.22)	558 (2.09)	592 (1.97)	650 (1.91)	**6a**	415 (4.58)	510 (3.41)	545 (3.25)	582 (3.32)	637 (3.08)
**3b**	417 (5.14)	515 (3.80)	549 (3.55)	592 (3.36)	648 (3.28)	**6b**	416 (5.42)	510 (4.23)	542 (3.66)	583 (3.86)	634 (3.69)
**3c**	418 (5.23)	516 (4.07)	555 (3.92)	593 (3.78)	652 (3.74)	**6e**	417 (5.59)	514 (4.35)	544 (3.68)	583 (3.97)	632 (3.21)

**Table 4:** Photophysical properties of the porphyrins in ethanol (λ_exc_ = 414 nm).

	Ethanol							D_2_O						
Compd	λ_f1_^a^ (nm)	λ_f2_^b^ (nm)	λ_f1_/λ_f2_	Φ_F_^c^	Φ_Δ_^d^	τ_F_ (ns)	τ_Δ_^e^ (µs)	λ_f1_^a^ (nm)	λ_f2_^b^ (nm)	λ_f1_/λ_f2_	Φ_F_^c^	Φ_Δ_^f^	τ_F_ (ns)	τ_Δ_^g^ (µs)
**1a**	645	712	0.75	0.10	0.74	10.0	15	−	−	−	−	−	−	−
**1b**	646	713	1.06	0.12	0.77	10.1	17	−	−	−	−	−	−	−
**1d**	663	716	1.29	0.02	0.05	3.2 (57%)	16	−	−	−	−	−	−	−
						8.5 (43%)								
**2a**	645	713	0.75	0.09	0.65	10.0	16	657	709	0.63	0.07	0.90	6.1	69
**2b**	656	716	1.27	0.12	0.82	8.9	16	655	709	0.59	0.07	0.93	4.8	66
**2d**	654	719	0.97	0	0	nd	nd	nd	nd	nd	0.01	nd	4.6	nd
**3a**	652	718	1.89	0.13	0.66	9.5	16	−	−	−	−	−	−	−
**3b**	650	717	1.7	0.09	0.57	10.1	17	−	−	−	−	−	−	−
**3c**	653	719	2.05	0.08	0.49	9.8	16	−	−	−	−	−	−	−
								−	−	−	−	−	−	−
**4a**	648	716	1.3	0.06	0.51	10	16	644	704	1.33	0.14	0.83	11.2	65
**4b**	649	716	1.43	0.07	0.54	9.4	15	660	719	1.45	0.01	nd	10.8	nd
**4c**	651	718	1.7	0.07	0.53	9.6	16	648	707	1.53	0.14	0.62	10.1	68
**5a**	648	714	1.26	0.14	0.74	8.7	15	−	−	−	−	−	−	−
**5b**	650	714	1.25	0.15	0.78	12.3	16	−	−	−	−	−	−	−
**5e**	649	711	0.62	0.10	0.81	9.7	15	−	−	−	−	−	−	−
								−	−	−	−	−	−	−
**6a**	639	709	1.75	0.13	0.63	12.7	17	635	702	2.66	0.15	0.60	17.4	60
**6b**	638	705	1.73	0.13	0.58	10.4	15	636	702	1.53	0.13	0.83	16.1	67
**6e**	639	704	0.39	0.11	0.78	11.9	15	638	698	0.39	0.09	0.86	14.9	69

^a^ Q (0,0). ^b^ Q (0,1). ^c^ TPP as standard (Φ_F_ (toluene) = 0.11) [68]. ^d^ TPP–COOH as standard (Φ**_Δ_** (EtOH) = 0.54) [69]. ^e^ Literature value~14.5 µs in EtOH [70]. ^f^ TMPyP as standard (Φ**_Δ_** (D_2_O) = 0.90) [68]. ^g^ Literature value~67 µs in D_2_O [71,72].

**Table 5:** Minimum bactericidal concentrations (MBCs) of the cationic porphyrins against *S. aureus*, *E. coli*, and *P. aeruginosa* after 20 h of white light irradiation (4.83 mW/cm², totaling 348 J/cm² fluence) at 37 °C. Each MBC is the concentration that inactivated at least 99.9% of bacterial cells, in comparison with the untreated control. Each experiment was performed six times.

MBC (μM)	2a	4a	6a
*S. aureus*	5.0	1.5	6.2
*E. coli*	18.0	2.5	40.0
*P. aeruginosa*	20.0	22.0	25.0

## Data Availability

Data available on request to florent.le-guern@uvsq.fr or corresponding authors.

## Supplementary Materials

The following are available online: Figures S1–S18. 1H NMR analyses, Figure S19. Fluorescence spectrum of **4d**.

## Sample Availability

Samples of the compounds are not available from the authors.
