# Supplementary material for: Photophysical and Bactericidal Properties of Pyridinium and Imidazolium Porphyrins for Photodynamic Antimicrobial Chemotherapy
Source: Molecules. 2021 Feb 20;26(4):1122. doi: 10.3390/molecules26041122 (PMC7924203; doi:10.3390/molecules26041122)
Supplement: Supplementary file 1 [file molecules-26-01122-s001.pdf]

Photophysical and bactericidal properties of pyridinium and imidazolium porphyrins for photodynamic antimicrobial chemotherapy

Florent Le Guern [1], Tan-Sothea Ouk [2], Issabayev Yerzhan [3], Yesmurzayeva Nurlykyz [3], Phillipe Arnoux [3], Céline Frochot [3], Stéphanie Leroy-Lhez [2], Vincent Sol [2,*]

[1] Université Paris-Saclay, UVSQ, CNRS, Institut Lavoisier de Versailles, 78035, Versailles, France
[2] Université de Limoges, Laboratoire PEIRENE, EA 7500, 123 Avenue Albert Thomas, 87060 Limoges Cedex, France.
[3] Université de Lorraine, Laboratoire Réactions et Génie des Procédés (LRGP), UMR 7274 CNRS, ENSIC, 1 rue Grandville, 54000 Nancy, France.
*Corresponding author: Tel.: +33(0)-5-5545-7490; fax: +33(0)-5-5545-7202; e-mail: vincent.sol@unilim.fr

Figure S1. 1H NMR analysis of 1a. 2
Figure S2. 1H NMR analysis of 1b. 2
Figure S3. 1H NMR analysis of 1d. 3
Figure S4. 1H NMR analysis of 2a. 3
Figure S5. 1H NMR analysis of 2b. 4
Figure S6. 1H NMR analysis of 2d. 4
Figure S7. 1H NMR analysis of 3a. 5
Figure S8. 1H NMR analysis of 3b. 5
Figure S9. 1H NMR analysis of 3c. 6
Figure S10. 1H NMR analysis of 4a. 6
Figure S11. 1H NMR analysis of 4b. 7
Figure S12. 1H NMR analysis of 4c. 7
Figure S13. 1H NMR analysis of 5a. 8
Figure S14. 1H NMR analysis of 5b. 8
Figure S15. 1H NMR analysis of 5e. 9
Figure S16. 1H NMR analysis of 6a. 9
Figure S17. 1H NMR analysis of 6b. 10
Figure S18. 1H NMR analysis of 6e. 10
Figure S19. Fluorescence spectrum of 4d in ethanol (λexc = 414 nm). The primary amine function does not lead to the quenching of the emission in this case. 11

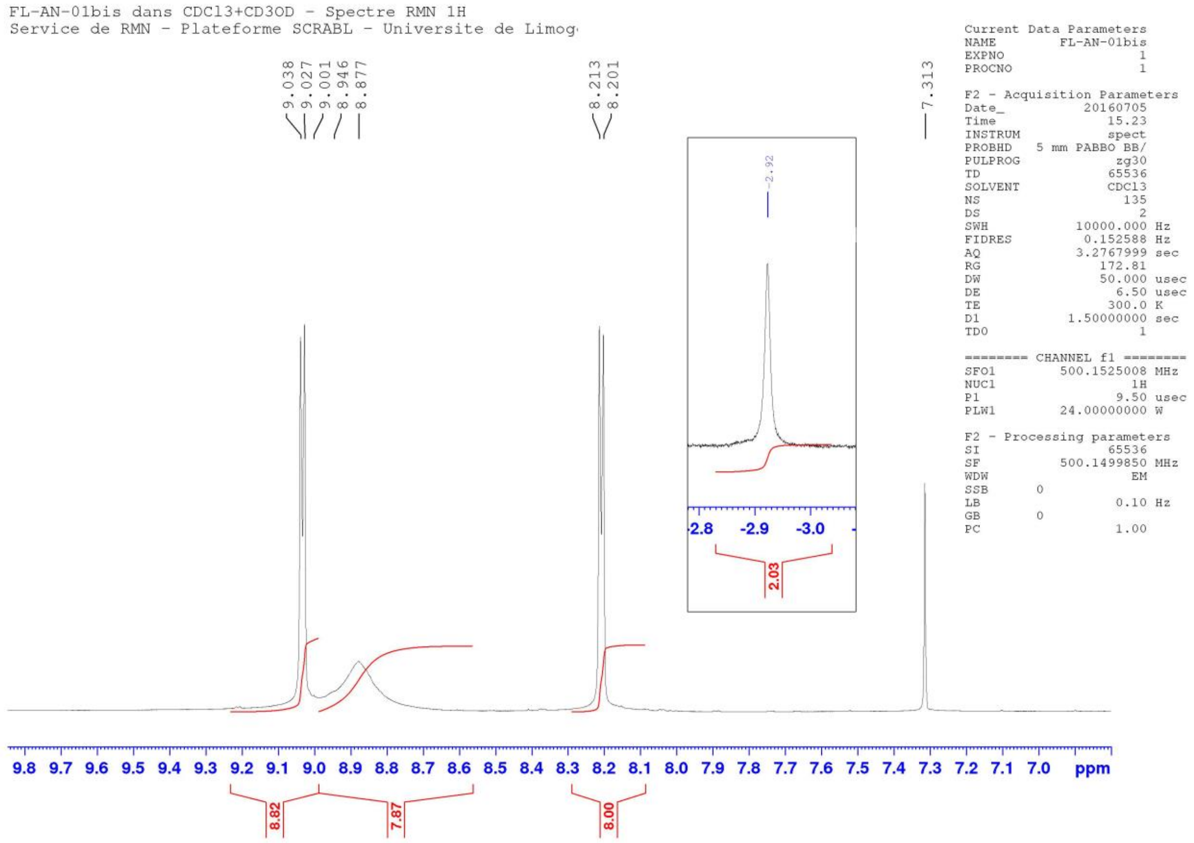

Figure S1. 1H NMR analysis of 1a.

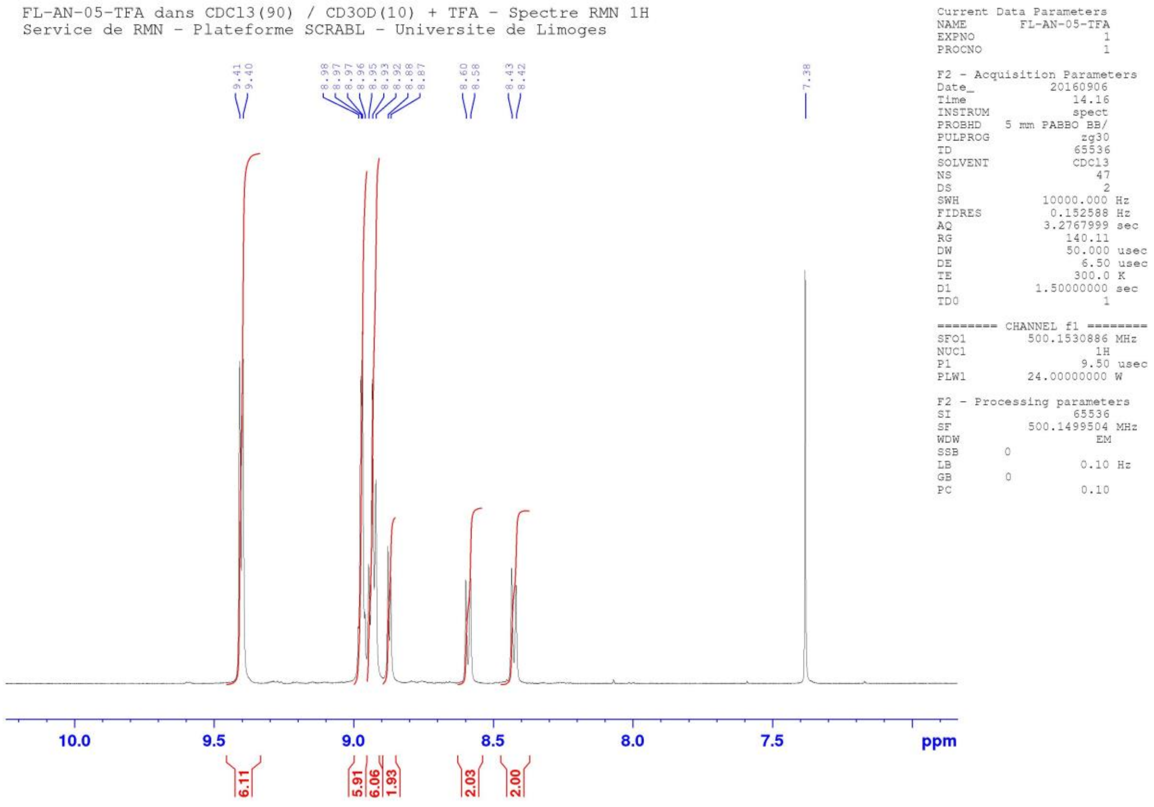

Figure S2. 1H NMR analysis of 1b.

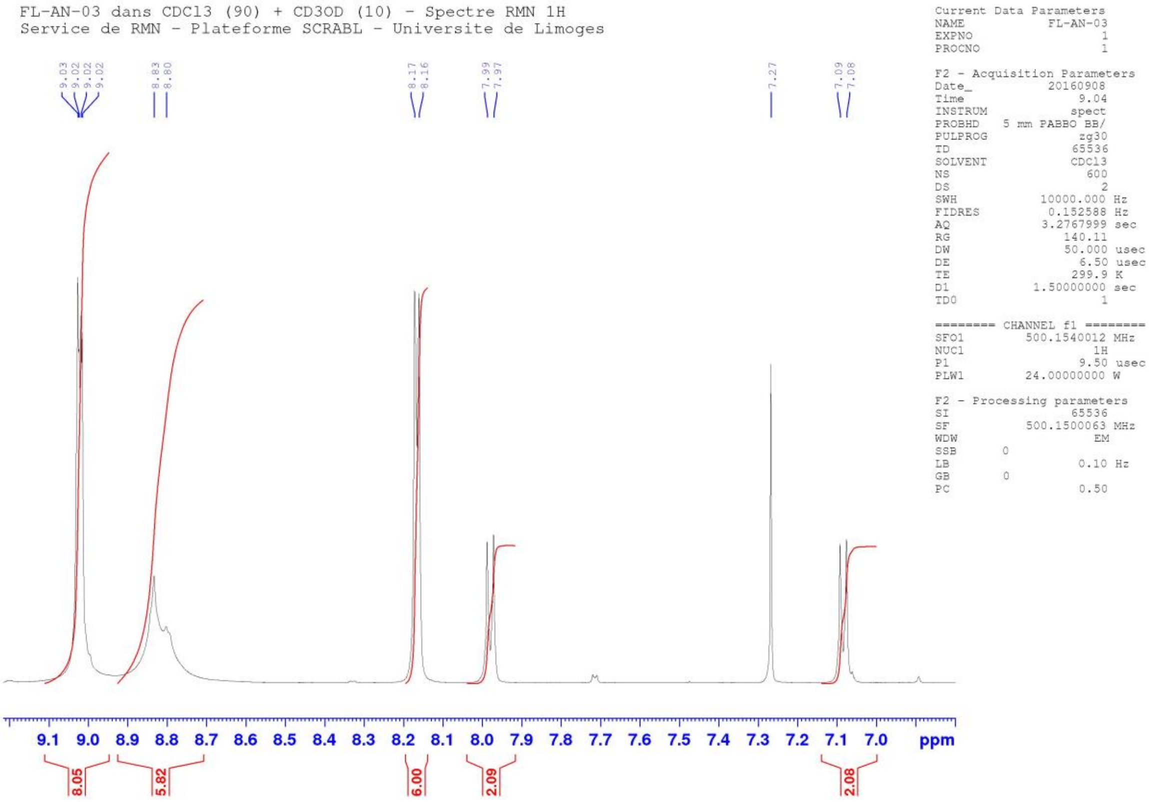

Figure S3. 1H NMR analysis of 1d.

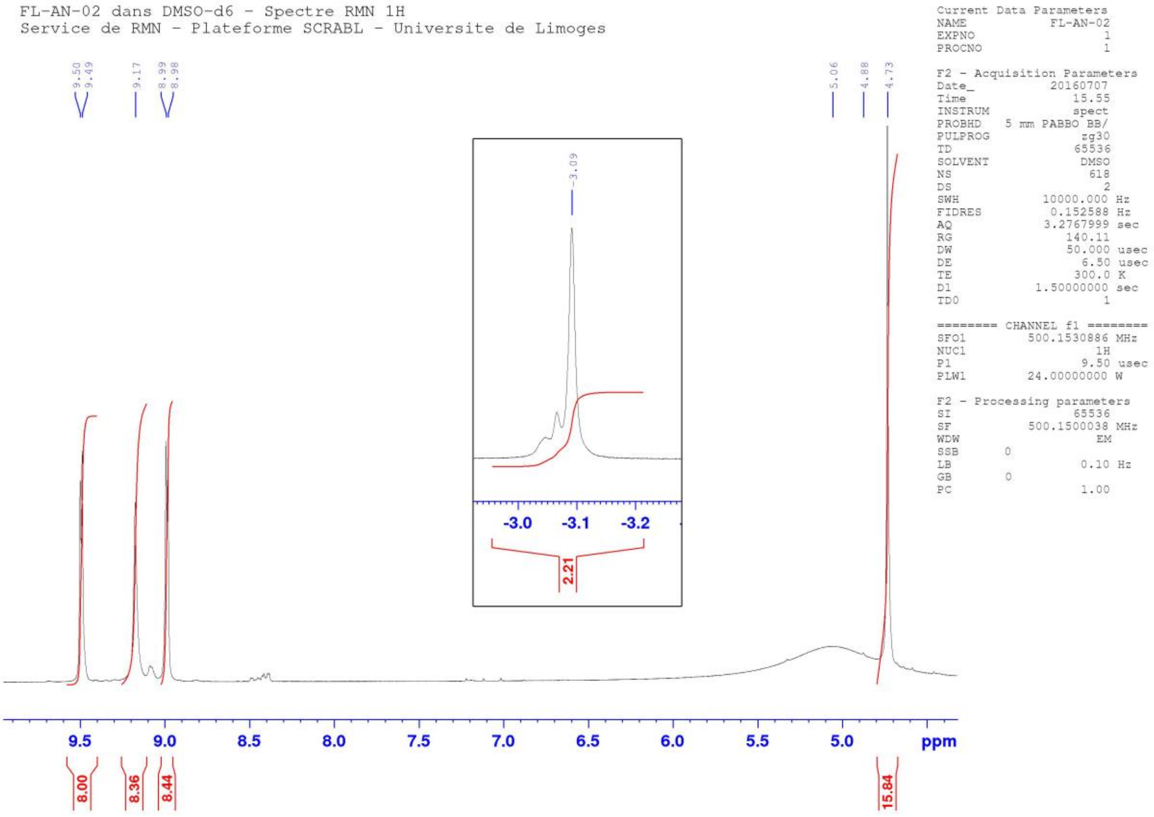

Figure S4. 1H NMR analysis of 2a.

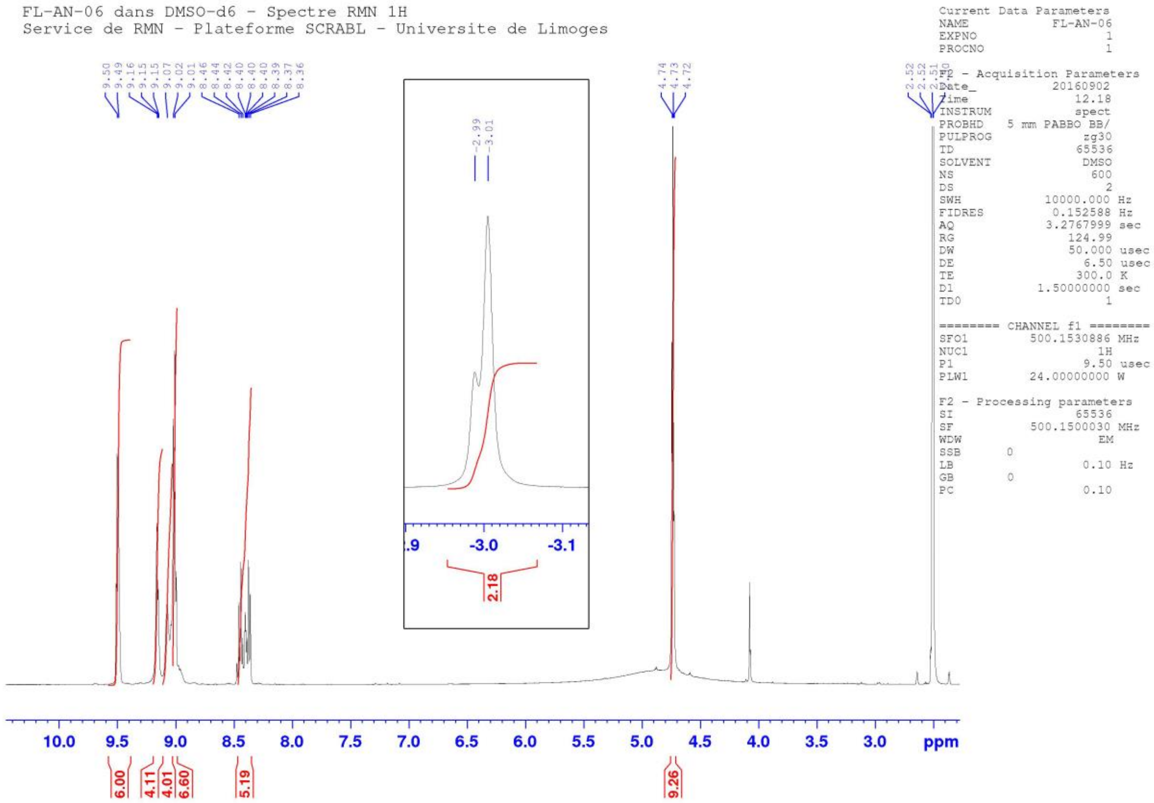

Figure S5. 1H NMR analysis of 2b.

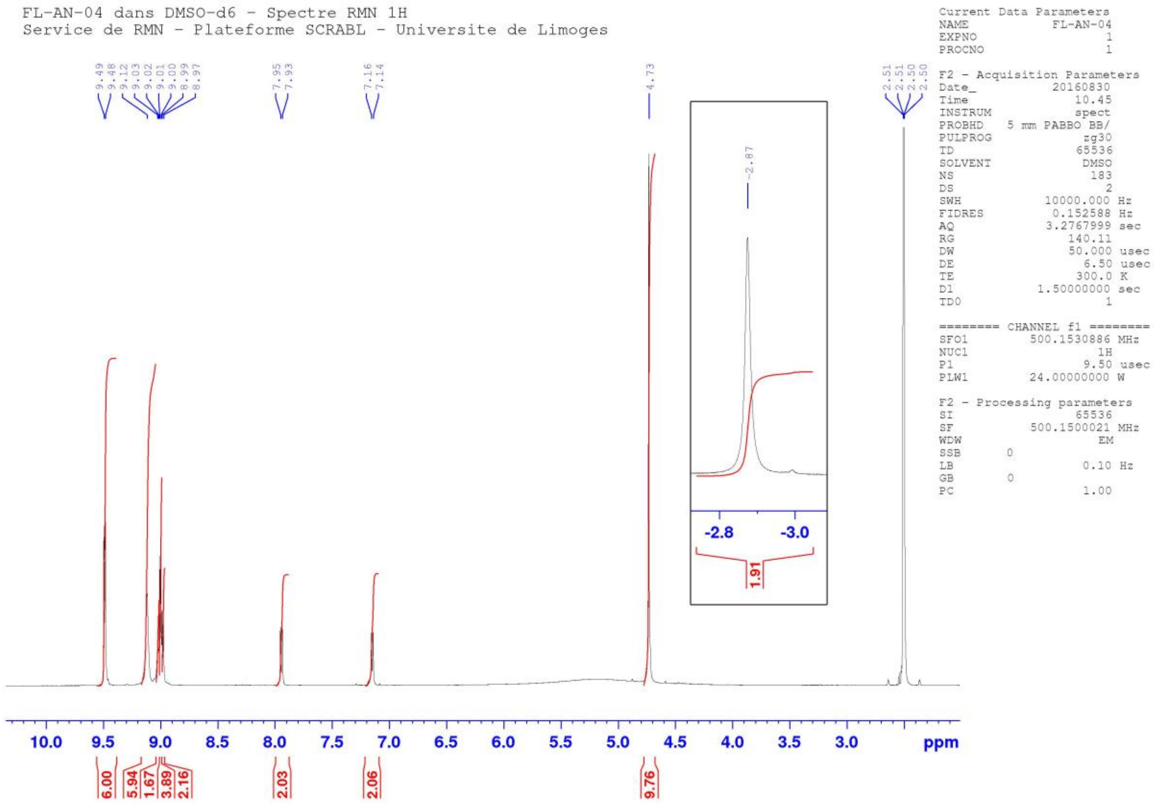

Figure S6. 1H NMR analysis of 2d.

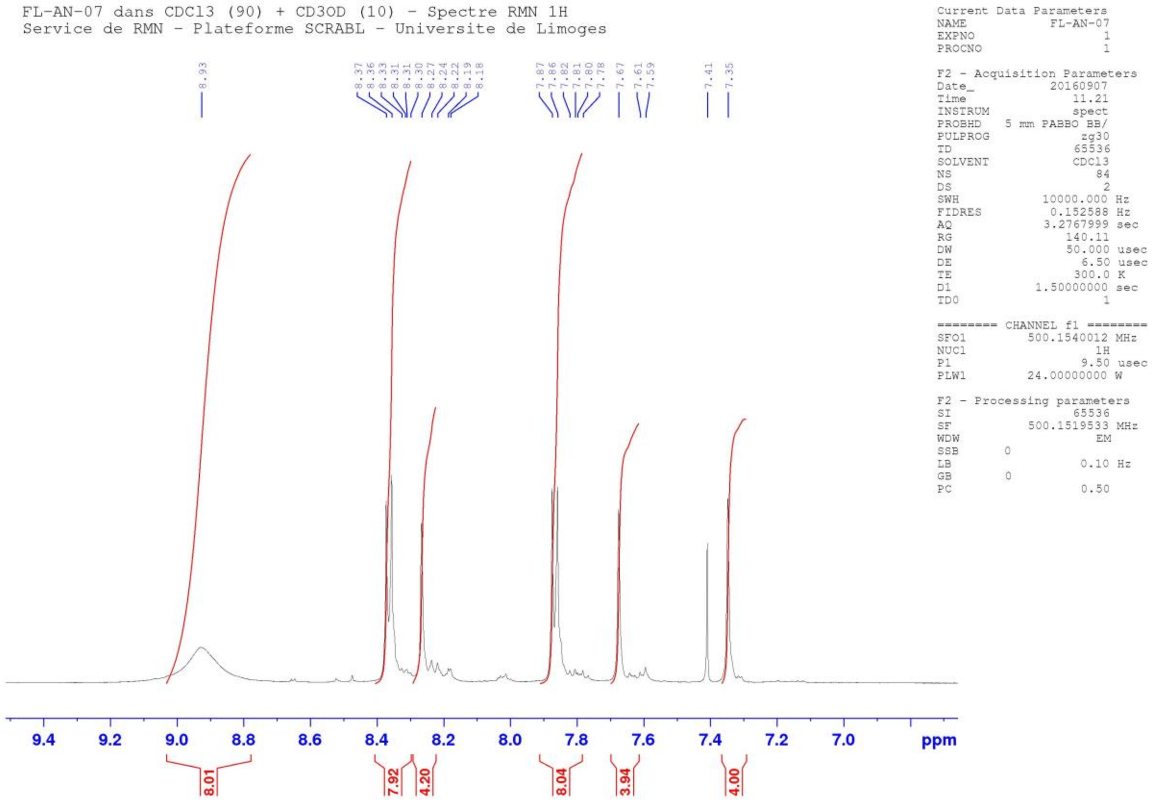

Figure S7. 1H NMR analysis of 3a.

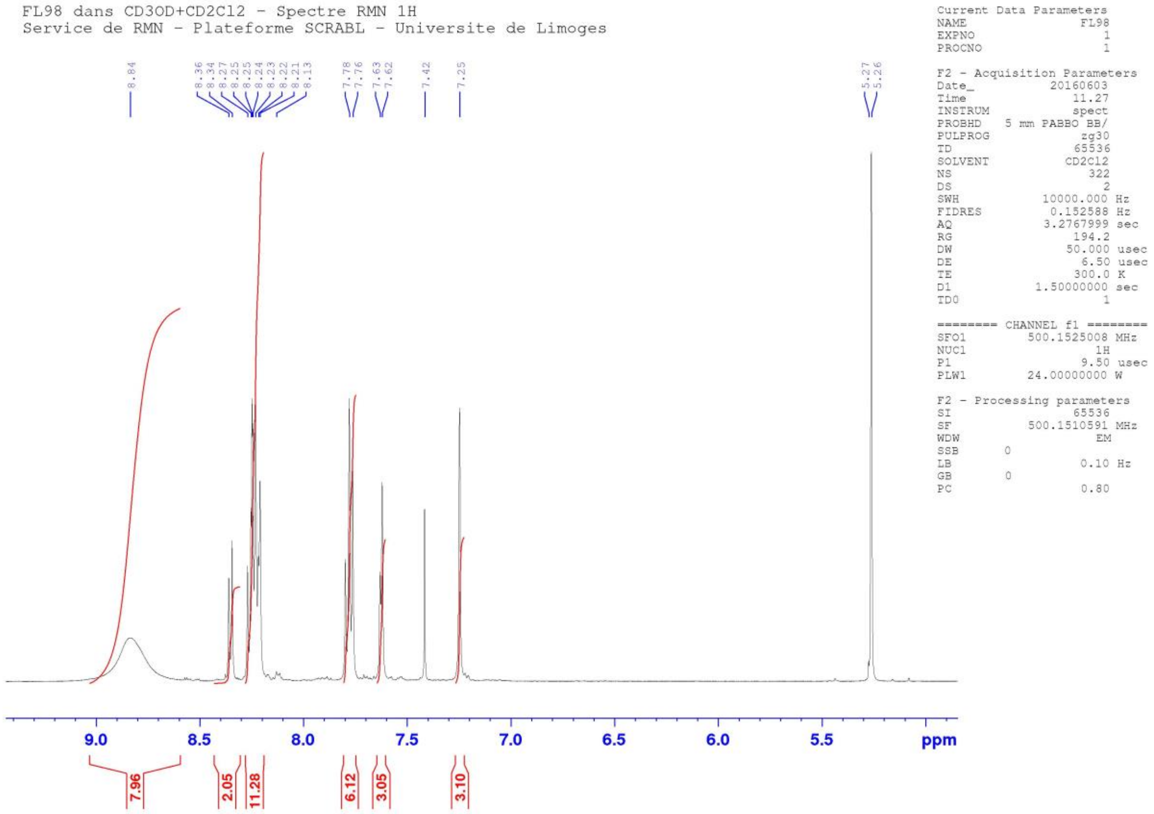

Figure S8. 1H NMR analysis of 3b.

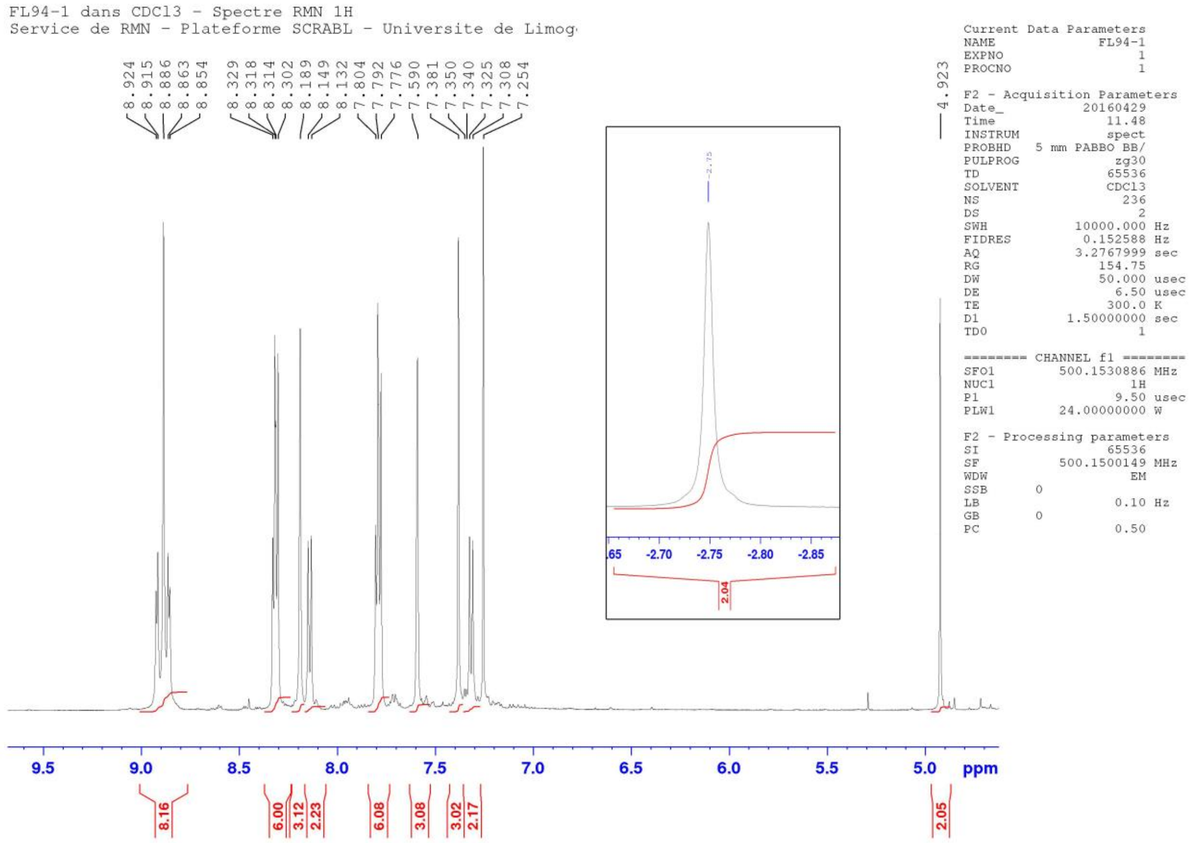

Figure S9. 1H NMR analysis of 3c.

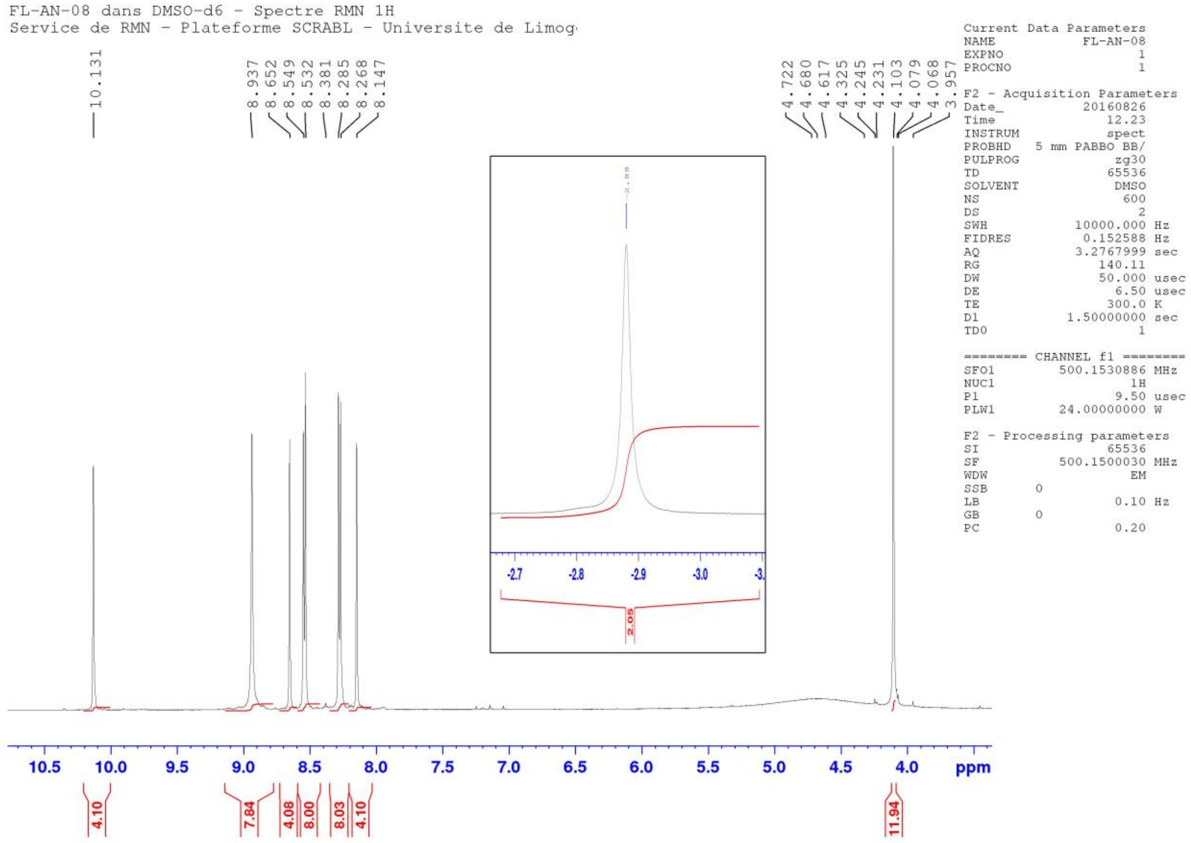

Figure S10. 1H NMR analysis of 4a.

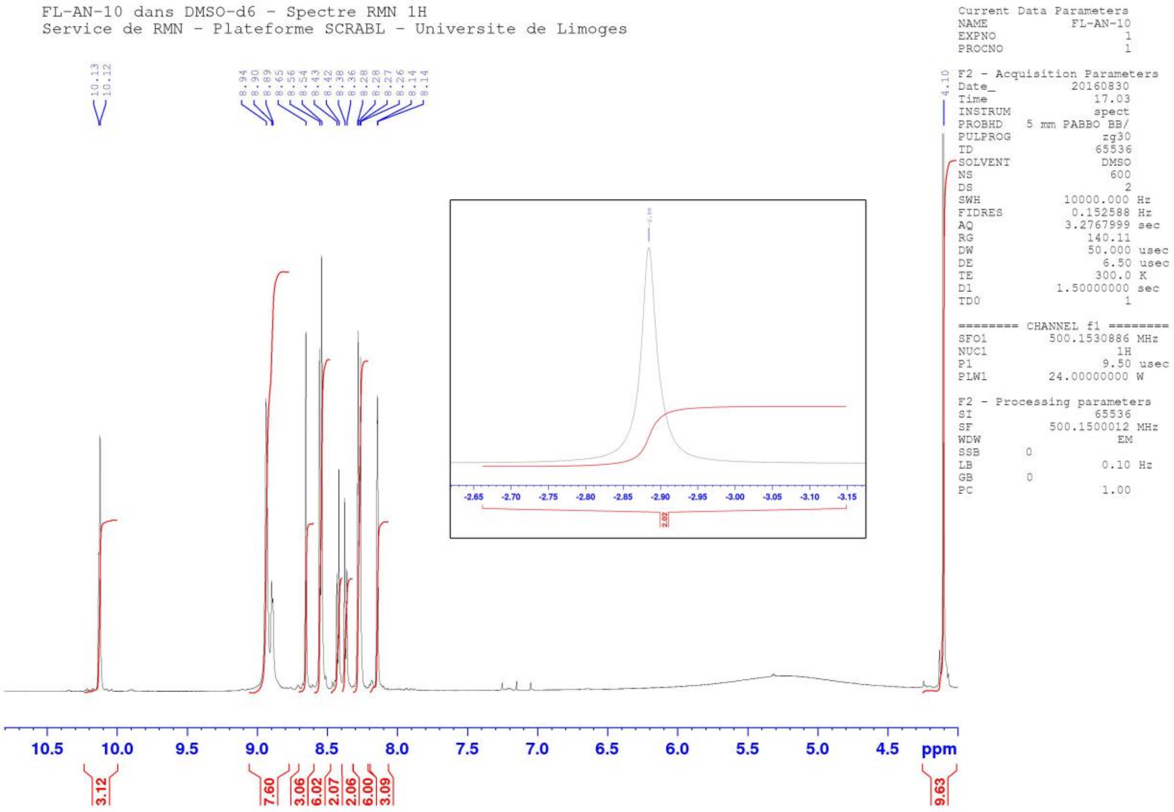

Figure S11. 1H NMR analysis of 4b.

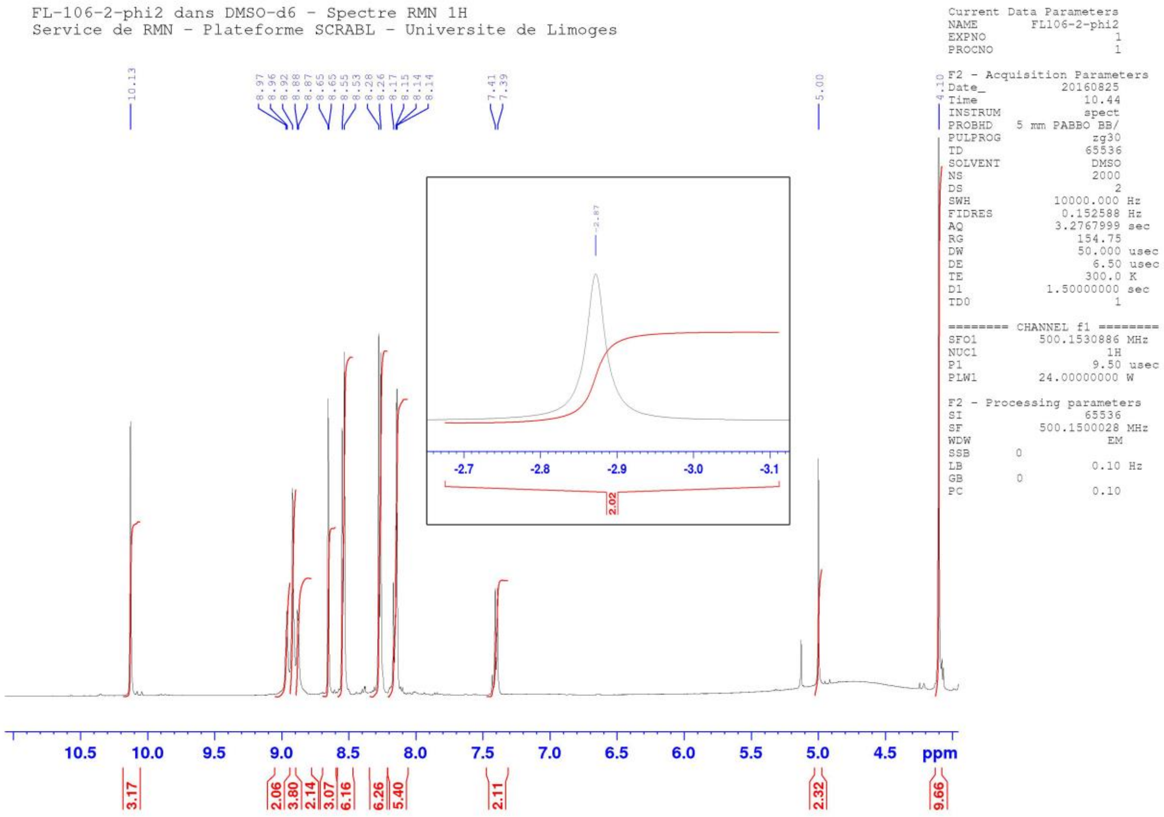

Figure S12. 1H NMR analysis of 4c.

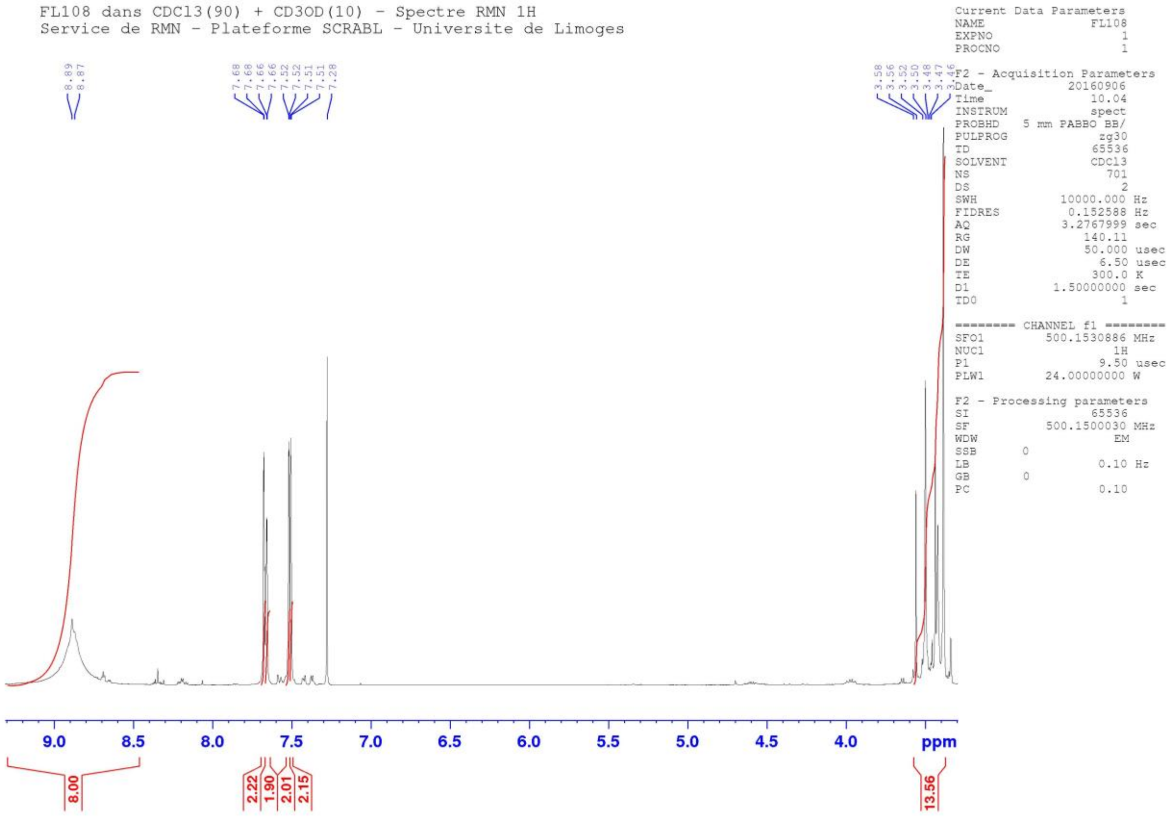

Figure S13. 1H NMR analysis of 5a.

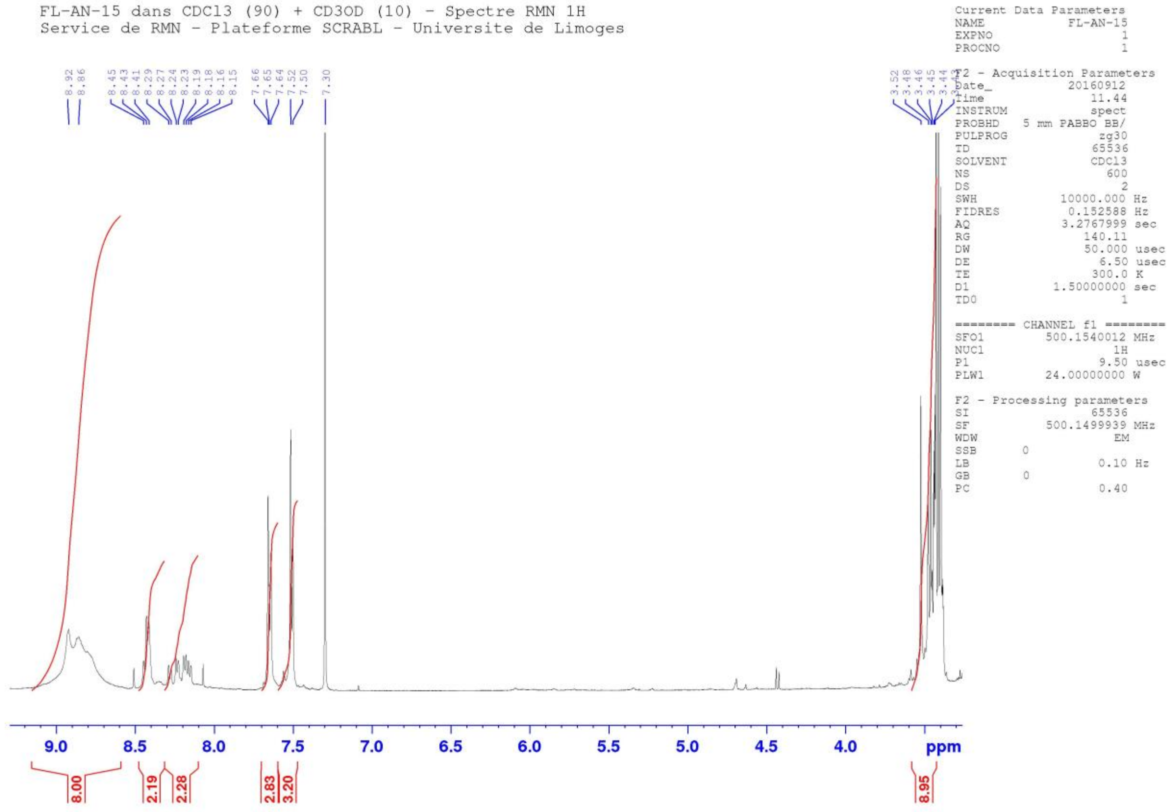

Figure S14. 1H NMR analysis of 5b.

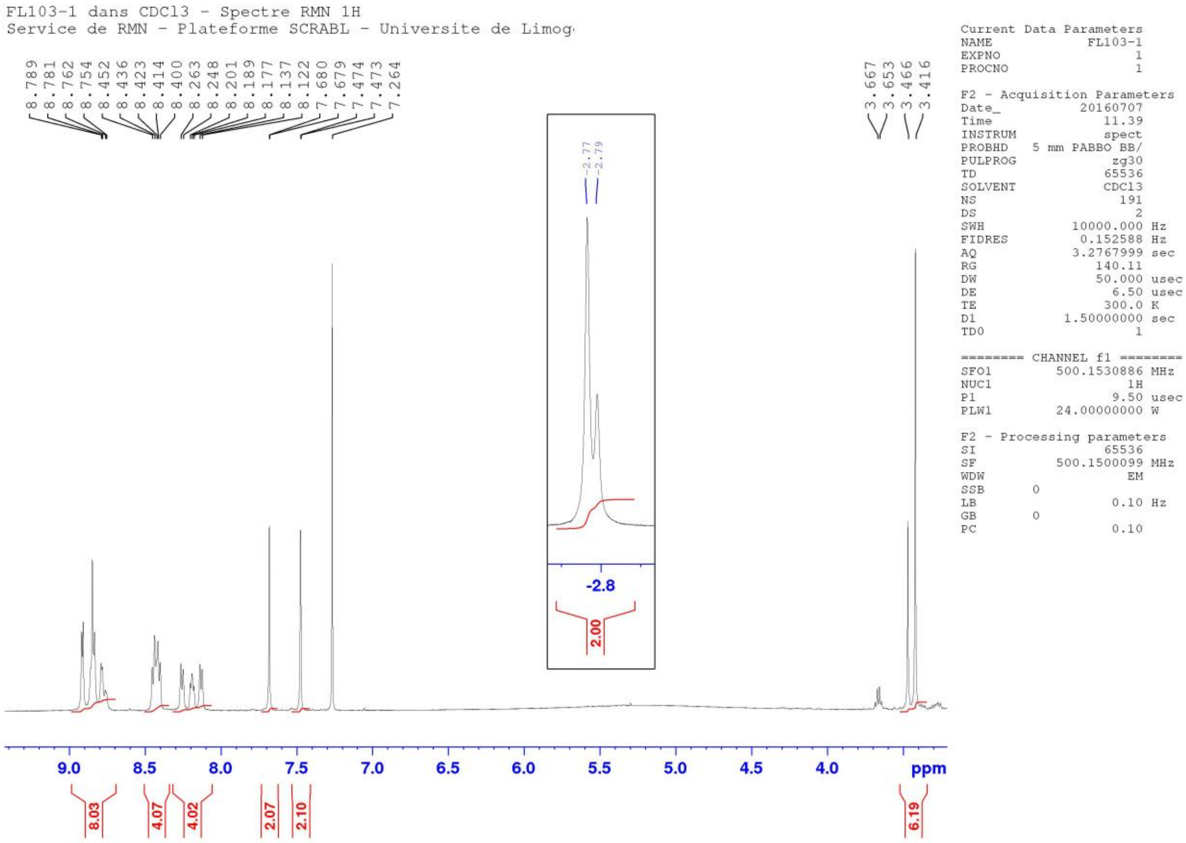

Figure S15. 1H NMR analysis of 5e.

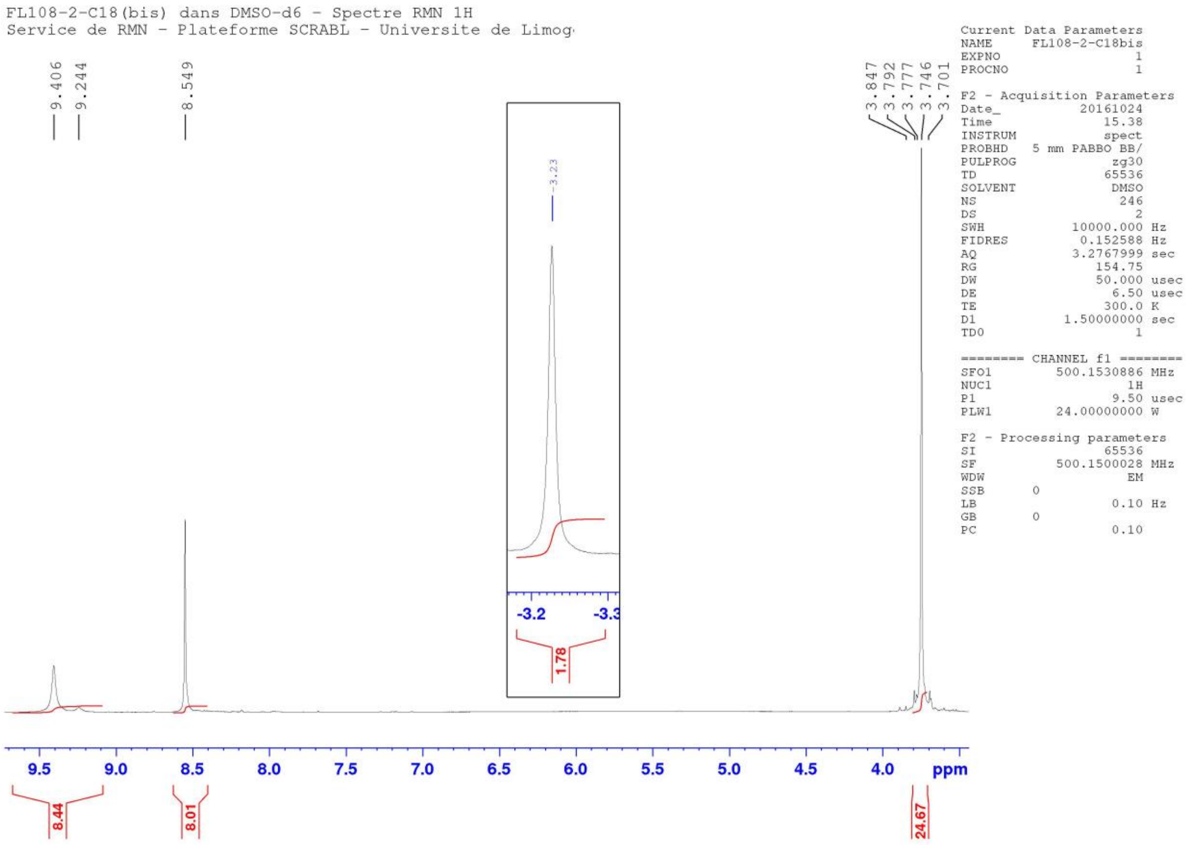

Figure S16. 1H NMR analysis of 6a.

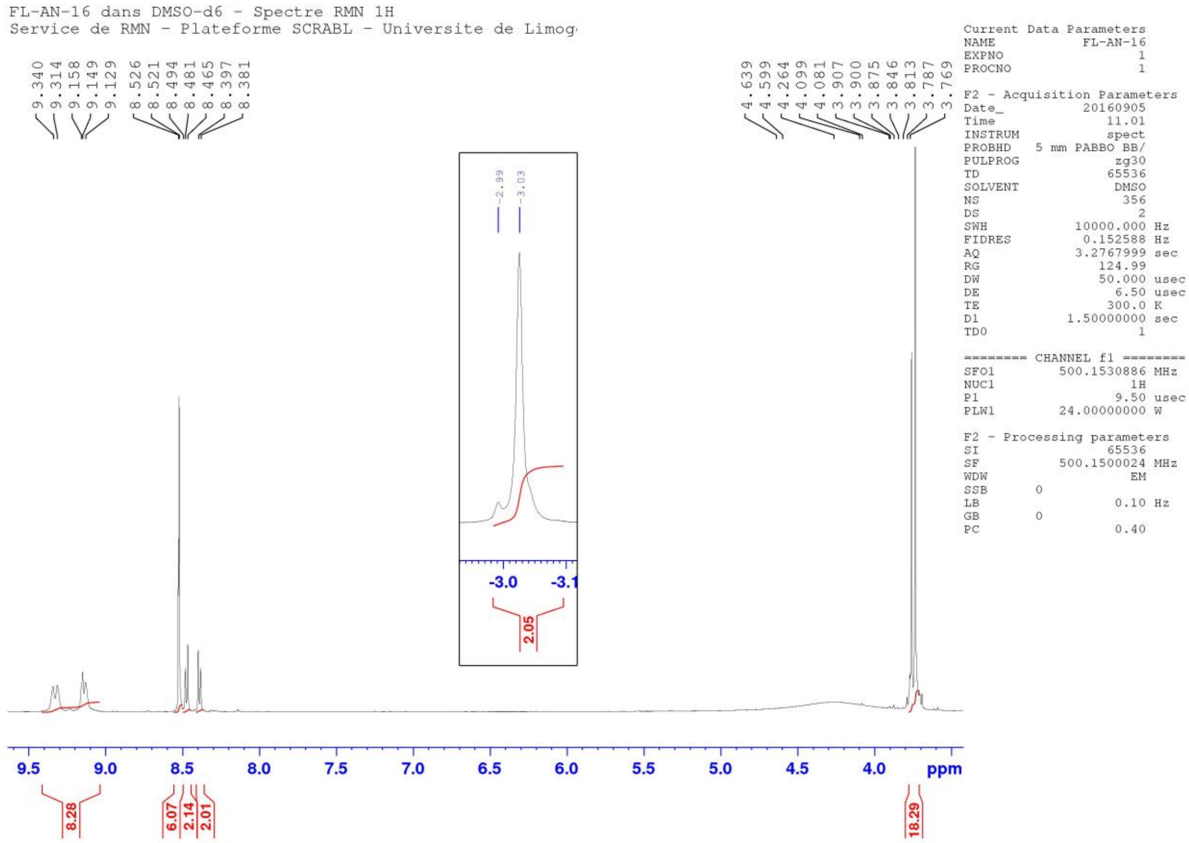

Figure S17. 1H NMR analysis of 6b.

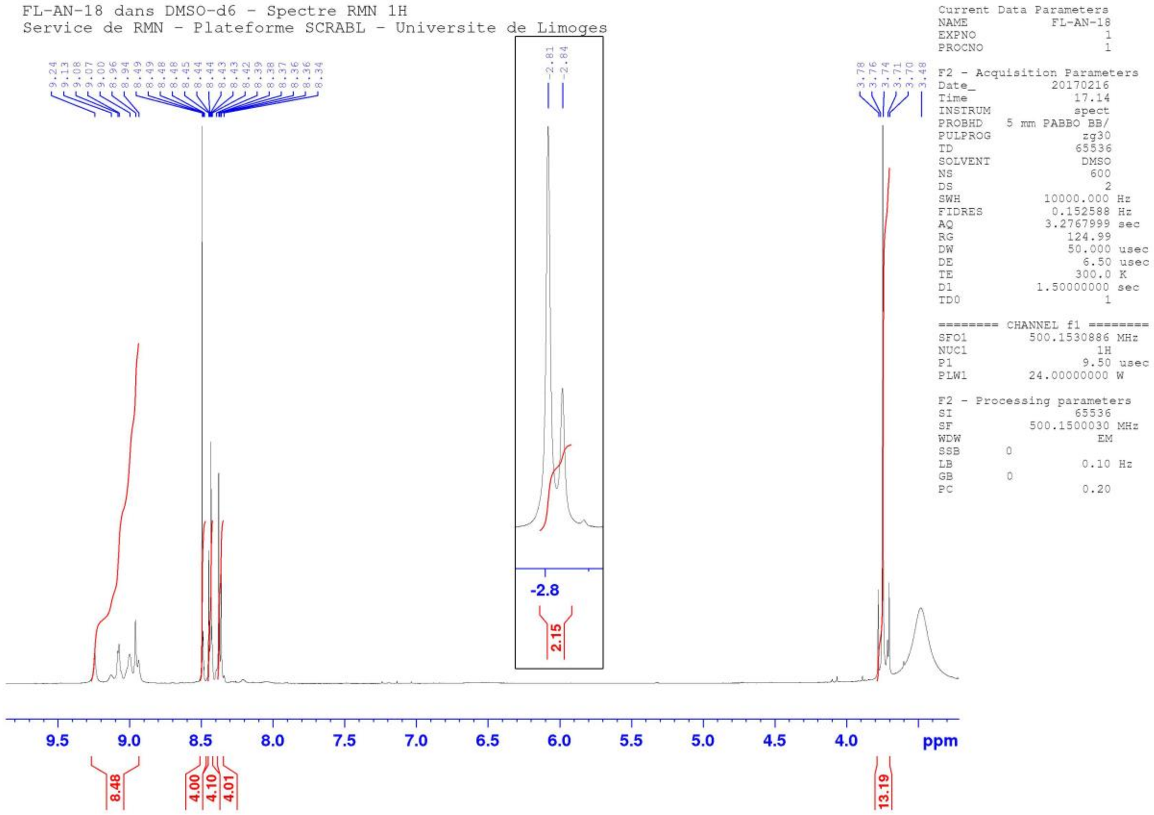

Figure S18. 1H NMR analysis of 6e.

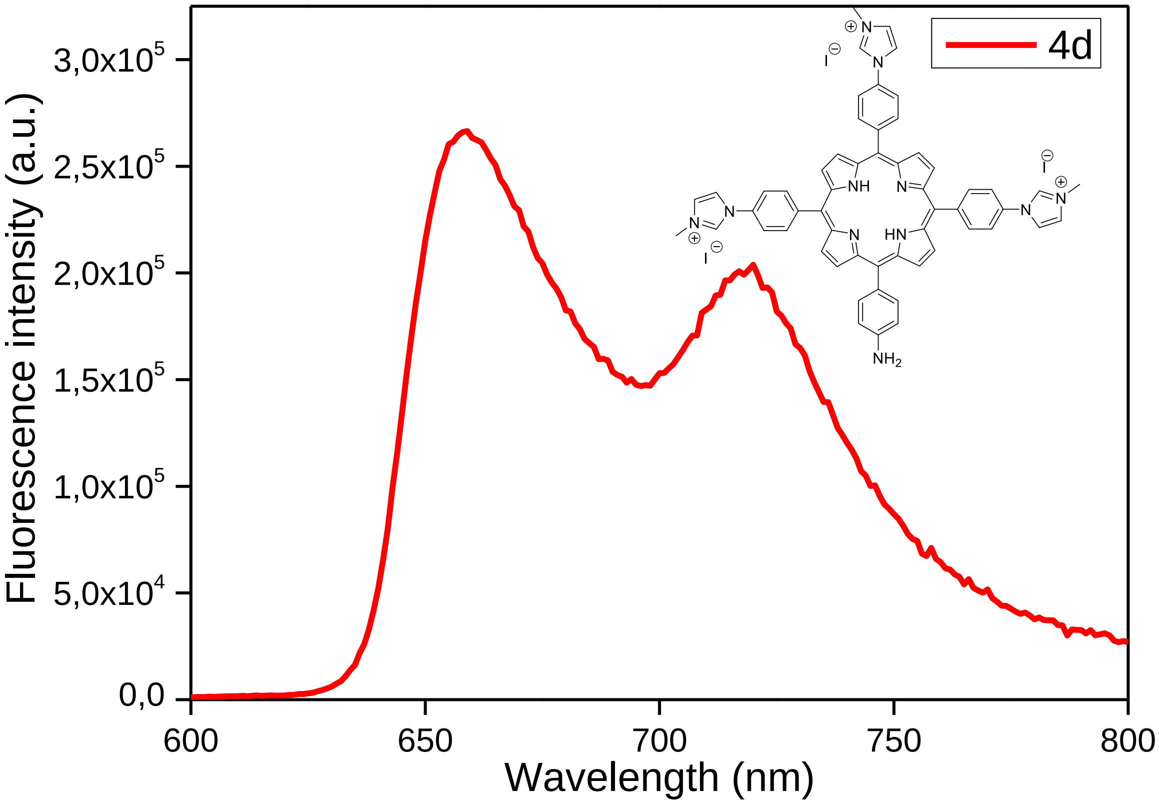

Figure S19. Fluorescence spectrum of 4d in ethanol (λexc = 414 nm). The primary amine function does not lead to the quenching of the emission in this case.
